# Arachidonate 15-lipoxygenase type B: Regulation, function, and its role in pathophysiology

**DOI:** 10.3389/fphar.2022.1042420

**Published:** 2022-11-09

**Authors:** Yvonne Benatzy, Megan A. Palmer, Bernhard Brüne

**Affiliations:** ^1^ Faculty of Medicine, Institute of Biochemistry I, Goethe University Frankfurt, Frankfurt, Germany; ^2^ Frankfurt Cancer Institute, Goethe University Frankfurt, Frankfurt, Germany; ^3^ German Cancer Consortium (DKTK), Partner Site Frankfurt, Frankfurt, Germany; ^4^ Fraunhofer Institute for Translational Medicine and Pharmacology ITMP, Frankfurt, Germany

**Keywords:** ALOX15B, lipoxygenase, PUFA, 15-HETE, lipid mediator, cancer, immunity, inflammation

## Abstract

As a lipoxygenase (LOX), arachidonate 15-lipoxygenase type B (ALOX15B) peroxidizes polyenoic fatty acids (PUFAs) including arachidonic acid (AA), eicosapentaenoic acid (EPA), docosahexaenoic acid (DHA), and linoleic acid (LA) to their corresponding fatty acid hydroperoxides. Distinctive to ALOX15B, fatty acid oxygenation occurs with positional specificity, catalyzed by the non-heme iron containing active site, and in addition to free PUFAs, membrane-esterified fatty acids serve as substrates for ALOX15B. Like other LOX enzymes, ALOX15B is linked to the formation of specialized pro-resolving lipid mediators (SPMs), and altered expression is apparent in various inflammatory diseases such as asthma, psoriasis, and atherosclerosis. In primary human macrophages, ALOX15B expression is associated with cellular cholesterol homeostasis and is induced by hypoxia. Like in inflammation, the role of ALOX15B in cancer is inconclusive. In prostate and breast carcinomas, ALOX15B is attributed a tumor-suppressive role, whereas in colorectal cancer, ALOX15B expression is associated with a poorer prognosis. As the biological function of ALOX15B remains an open question, this review aims to provide a comprehensive overview of the current state of research related to ALOX15B.

## Introduction

Arachidonate 15-lipoxygenase type B (ALOX15B) is one of two described lipoxygenases that catalyze the peroxidation of arachidonic acid (AA) with positional specificity at carbon atom 15 (C_15_) (Brash et al., 1997). Lipoxygenases (LOXs) are non-heme iron containing dioxygenases that convert polyenoic fatty acids (PUFAs) to the corresponding short-lived hydroperoxy derivatives (Kühn et al., 2015). These fatty acid hydroperoxides then undergo a subsequent reduction to lipid alcohols or conversion to secondary oxygenation products. As biologically active lipid mediators, the produced fatty acids are involved in a wide range of acute and chronic pathological conditions (Kühn et al., 2015). Depending on the metabolite, its effect and implication can be ambiguous: anti-inflammatory, pro-resolving, pro-inflammatory (Fogh et al., 1988), as well as pro- or anti-tumorigenic (Vaezi et al., 2021). Although initially classified according to their positional specificity of AA oxygenation, the six functional human LOX enzymes ALOX15B, ALOX15, ALOX5, ALOX12, ALOX12B, and ALOXE3 can also oxygenate other PUFA substrates i.e.; linoleic acid (LA), docosahexaenoic acid (DHA), and eicosapentaenoic acid (EPA). With the exception of the *ALOX5* gene, which maps to the long arm of chromosome 10 (10q11.21), all other functional LOX genes are located in a common gene cluster on the short arm of chromosome 17 (Kühn et al., 2018). Although differing mainly in their tissue expression and positional specificity of PUFA oxygenation, all mammalian LOX enzymes perform non-redundant biological functions. In particular, the LOX enzymes ALOX5 and ALOX15 are well studied for their singular and collective synthesis of bioactive lipid mediators, such as hydro(pero)xy fatty acids (Kühn 1996), AA-derived leukotrienes (LT) (Savari et al., 2014) and lipoxins as well as EPA- and DHA-derived resolvins (Rv) (Schebb et al., 2022). Nevertheless, ALOX15B has also been implicated in the formation of distinct pro-resolving mediators, including resolvin RvD5 (Perry et al., 2020b).

ALOX15B was first described in 1997 as a previously unrecognized second 15-lipoxygenase with expression in human skin, prostate, lung and cornea (Brash et al., 1997) (for more details on tissue expression see Table 1 and *in vitro* expression Table 2). Brash et al. (1997) reported regiospecific and *S*-stereospecific oxygenation of AA to the corresponding fatty acid hydroperoxide 15(*S*)-hydroperoxyeicosatetraenoic acid (15(*S*)-HpETE), which was detected as the reduced hydroxide 15-hydroxyeicosatetraenoic acid (15(*S*)-HETE) (Brash et al., 1997). Shortly after, a mouse homolog structurally close to human ALOX15B was identified as the murine enzyme Alox8 (often also stated as mouse Alox15b or 8-LOX) (Jisaka et al., 1997; Brash et al., 1999). Alox8 shares 78% sequence identity with human ALOX15B but both enzymes differ with respect to their regiospecificity of AA oxygenation. In contrast to ALOX15B, Alox8 introduces molecular oxygen at C_8_ of AA, therefore catalyzing the formation of 8-HETE (Brash et al., 1999; Jisaka et al., 2000). Intriguingly, the difference in substrate regiospecificity is mediated by the substitution of only two amino acids between Alox8 and ALOX15B, namely exchange of Tyr^603^/His^604^ in Alox8 towards Asp^602^/Val^603^ in ALOX15B and their substitution converts the positional specificity of AA oxygenation from C_8_ to C_15_ and vice versa (Jisaka et al., 2000). Interestingly, inducible expression of both enzymes attenuated cell growth in keratinocytes (Schweiger et al., 2007). Recent data from *Alox15b* knock-in mice, humanized by mutation of the identified amino acid pair in Alox8, show that the hematopoietic system including erythrocyte number, hematocrit and hemoglobin is impaired in male mice. Also, this is paralleled by a premature growth arrest (Schäfer et al., 2022). These mice provide a rare insight into the biological function of human ALOX15B and mouse Alox8 *in vivo*, as there are no commercially available Alox8-deficient mice to date. How ALOX15B is implicated in the regulation of erythropoiesis is unknown so far, but apart from this, ALOX15B is linked to a variety of pathological states. With this review, we aim to summarize the existing findings on the enzymatic and biological function of ALOX15B and cast light on its implication in inflammatory diseases and cancer.

**TABLE 1:** Tissue expression of ALOX15B.

Tissue	Cell type	Cellular location	RNA (R) Protein (P)	References
Bladder	Epithelial		R	Subbarayan et al. (2005)
Breast	Epithelial, endothelial, vascular smooth muscle	Cytoplasmic and nuclear	R P	Subbarayan et al. (2005) and Jiang et al. (2006)
Joint	Chondrocytes		R P	Lin et al. (2021)
Endometrium	Epithelial and stromal	Cytoplasmic	P	Russell et al. (2011)
Esophageal	Epithelial	Cytoplasmic	R P	Xu et al. (2003)
Gastrointestinal system (small intestine, colon, rectum)	Enteric glial cells and neurons	Cytoplasmic	R P	Pochard et al. (2016)
Heart	Cardiac fibroblasts		R P	Sandstedt et al. (2018)
Kidney			P	Daurkin et al. (2011)
Lung	Type II pneumocytes	Cytoplasmic and nuclear	R P	Gonzalez et al. (2004) and Kilty et al. (1999)
Lymph node			R	Kilty et al. (1999)
Placenta (amnion)	Fibroblast		R P	Zhang et al. (2022)
Prostate	Epithelial	Cytoplasmic and nuclear	R P	Kilty et al. (1999), Shappell et al. (1999), Bhatia et al. (2003), Suraneni et al. (2014), and Subbarayan et al. (2005)
Skin and hair follicle	Epithelial	Cytoplasmic and plasma membrane	R P	Brash et al. (1997), Simard-Bisson et al. (2018), Kilty et al. (1999), and Shappell et al. (2001a)
Spinal cord			R	Kilty et al. (1999)

**TABLE 2:** Primary cells and cell lines expressing ALOX15B.

Origin tissue	Cell line	Cell type	Origin	RNA (R)	Reference
				Protein (P)	
Breast	HMEC	Epithelial	Primary	R P	Subbarayan et al. (2005)
	MCF10A		Fibrotic disease	R	Wang et al. (2017)
	MDA-MB-231		Adenocarcinoma	R	
Bladder (Ureter)	SV-HUC	Epithelial	SV-40 immortalized	R P	Subbarayan et al. (2005)
Colon	NCM460	Epithelial	Normal tissue	R P	Yuan et al. (2020)
	SW480		Adenocarcinoma	R P	Yuan et al. (2020), Subbarayan et al. (2005)
Lymph node metastasis	SW620	Epithelial	Adenocarcinoma	R P	Yuan et al. (2020), Subbarayan et al. (2005)
Colon	Caco-2	Epithelial	Adenocarcinoma	R	Pochard et al. (2016)
	EGC	Glial	Primary	R P	
Esophageal	EEC	Epithelial	Primary	R P	Xu et al. (2003)
Leukocytes	Human monocyte derived	Macrophages	Primary	R P	Snodgrass et al. (2018)
	Lung derived			R P	Abrial et al. (2015)
	TAMs			R P	Daurkin et al. (2011), Reinartz et al. (2016)
Liver	HepG2	Epithelial	Hepatocellular carcinoma	R P	Subbarayan et al. (2005)
	Hep3B				
Bronchial	NHBE	Epithelial	Primary	R P	Subbarayan et al. (2005), Shum et al. (2022)
	BET-1A		SV40 immortalized	R P	Li et al. (2019)
	NCI-H23		Adenocarcinoma	R P	
	A549		Adenocarcinoma	R P	Yang et al. (2018)
	CFBE41O-		Transformed cystic fibrosis	P	Shum et al. (2022)
Tracheo-bronchial	NHTBE	Epithelial	Primary at air liquid interphase	R	Kilty et al. (1999)
Placenta	Amnion	Fibroblast	Primary	R P	Zhang et al. (2022)
Prostate	NHP (PrEC)	Epithelial	Primary	R P	Subbarayan et al. (2005)
Pancreas	Panc-1	Epithelial	Epithelioid Carcinoma	P	Subbarayan et al. (2005)
Skin	NHEK	Epithelial	Primary	R P	Subbarayan et al. (2005), Hellmann et al. (2018)
	MeWo	Melanocyte	Malignant melanoma	R P	Subbarayan et al. (2005)
	3S5			R P	
Unclear	MDA-MB-435	Melanocyte	Originally thought to be breast carcinoma, however, is reassigned as melanoma	P	Nony et al. (2005), Subbarayan et al. (2005), Wang et al. (2017)

## Structural organization of ALOX15B

**FIGURE 1:**
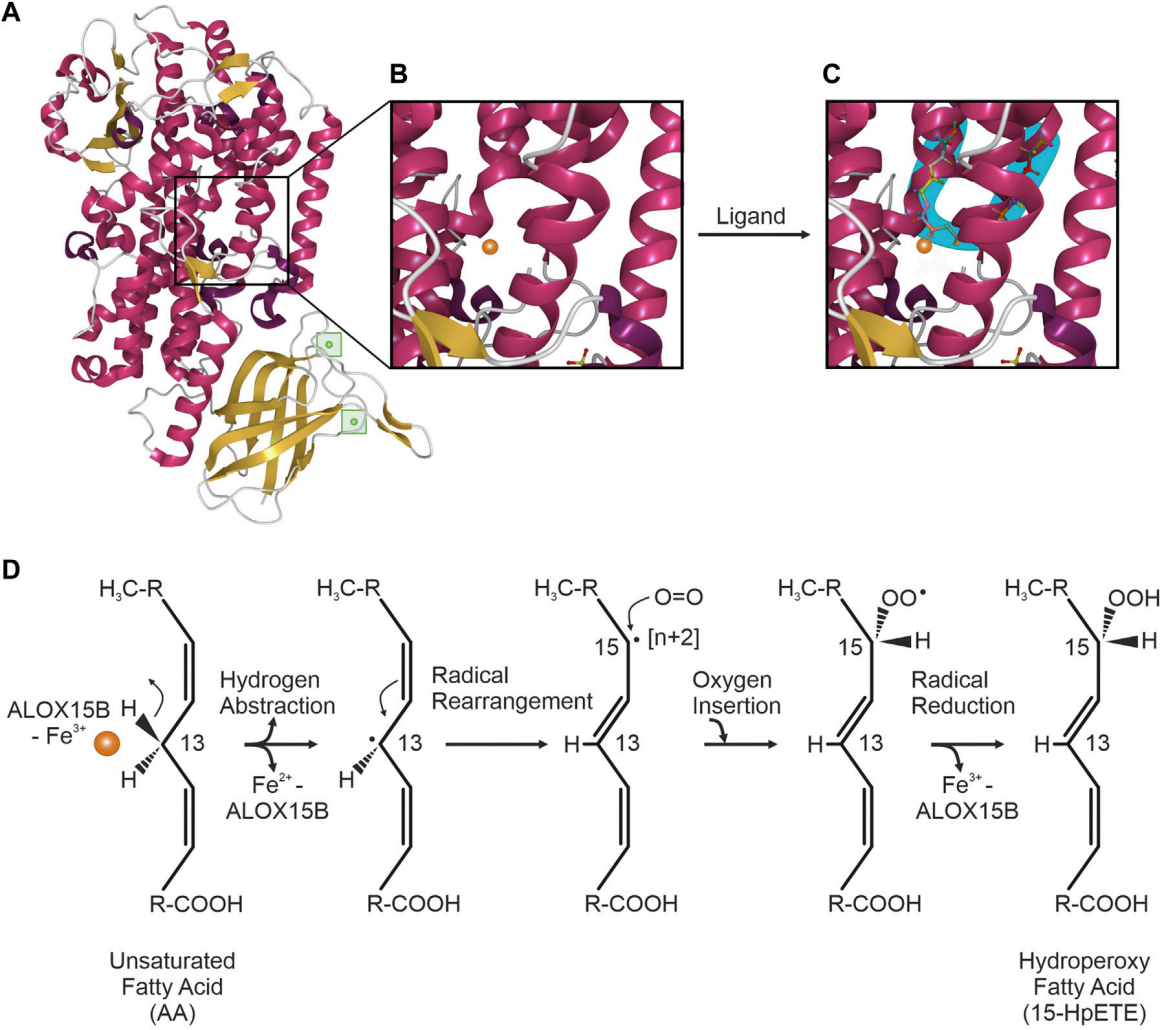
ALOX15B crystal structure and mechanism of fatty acid oxygenation. **(A)** Crystal structure of ALOX15B with C-terminal α-helical domain (red) and β-sheets (yellow). Inside the N-terminal 'β-barrel’, two Ca^2+^-binding sites (green) allow membrane binding of ALOX15B. **(B)** Zoom-in into the putative substrate-binding pocket inside the α-helical domain, containing a non-heme iron (orange). **(C)** Ligand binding to the U-shaped substrate binding channel (blue) places the respective pentadiene close to the reactive iron (orange). Crystal structure was obtained and modified based on the RCSB PDB (rcsb.org) dataset 4NRE (DOI: 10.2210/pdb4NRE/pdb), deposited by Kobe et al. (2014) and visualized with Mol* Viewer (Sehnal et al., 2021). **(D)** ALOX15B-catalyzed oxygenation of an unsaturated fatty acid [example attack here with arachidonic acid (AA)] is initiated by the hydrogen abstraction of a *cis*,*cis*-1,4-pentadiene (at C_13_ of AA) promoted through the ferric (Fe^3+^) iron (orange) of the ALOX15B catalytic center, leading to an inactive enzyme (Fe^2+^). Upon rearrangement of the radical from C_13_ to C_15_ [+2], molecular oxygen is inserted antarafacially to the hydrogen abstraction at C_15_. Reduction of the resulting peroxyl-radical is mediated through electron transfer from Fe^2+^ and is accompanied with the re-oxidation to Fe^3+^, thus restoring enzyme activity. Finally, a fatty acid hydroperoxide is formed through the protonation of the resulting peroxy-anion.

Mammalian LOX enzymes are single polypeptide chain proteins that fold into a two-domain structure with a C-terminal α-helical domain, containing a non-heme iron carrying putative substrate-binding pocket and an N-terminal 'β-barrel’ (Figures 1A,B). The latter functions in the acquisition of the PUFA substrate (Figure 1C) and the calcium (Ca^2+^)-dependent membrane binding *via* a polycystin-1-lipoxygenase α-toxin (PLAT) domain (Brinckmann et al., 1998; Kühn et al., 2015). Crystal structure analysis indicated that ALOX15B obtains the typical LOX fold, harboring an amino-terminal PLAT domain with two Ca^2+^-binding sites and a large α-helical catalytic domain (Kobe et al., 2014) (Figure 1A). Furthermore, the ALOX15B crystal structure revealed an ALOX15B unique hydrophobic hairpin, which apparently helps to position the enzyme at the phospholipid bilayer upon Ca^2+^-binding, thereby allowing it to function as a membrane anchor and supporting the accessibility to membrane-bound substrates (Kobe et al., 2014). In agreement, further reports of crystal structure analysis and ALOX15B expressing cell lines demonstrated a Ca^2+^-dependent association of human ALOX15B with the membrane fraction (Kilty et al., 1999; Bender et al., 2016) and described a Ca^2+^-stabilized putative membrane insertion loop, which was found to project from the amino-terminal membrane binding domain (Bender et al., 2016). Using cytosol-free, recombined fractions from LOX-absent cell lines, the presence of Ca^2+^ led to translocation of recombinant ALOX15B to the membrane fraction, which renders dependence on a membrane-docking protein unlikely (Kilty et al., 1999). However, Western blotting of membrane and cytosol fractions of ALOX15B-expressing cell lines demonstrated the dominant cytosolic location of ALOX15B in resting cells (Kilty et al., 1999).

Also, in normal human prostate cells (NHP) ALOX15B was found to be predominantly localized in the cytosol, while some protein was detected in the nucleus or associated with cytoskeletal proteins, microsomes, and membranes (Bhatia et al., 2003). Localization in the nucleus may suggest a non-enzymatic function of the protein as proposed for other LOX isoforms (Kreiß et al., 2022). Interestingly, alternative splice variants of ALOX15B have not been described to localize to the nucleus (Bhatia et al., 2003) and demonstrated very little AA metabolizing activity (Tang et al., 2007). Potentially, nuclear ALOX15B enzyme behaves comparable to other nuclear LOX isoforms in producing ligands for nuclear receptors.

In contrary to previous studies, using nanodisc-associated ALOX15B, kinetics of hydrogen/deuterium exchange indicated no structural changes of the PLAT domain upon Ca^2+^-binding, only upon ligand binding. It was proposed that the hydrophobic PLAT loop rather mediates a transient than a deep and stable insertion into the membrane. The authors proposed that Ca^2+^-binding to the PLAT domain mediates membrane association more likely through the addition of a positive charge to the PLAT domain, thereby coordinating an association with phosphatidylserine, than changing the structure and dynamics of the protein (Droege et al., 2017). This finding was supported by data of former work with nanodisc-associated ALOX15B, where no “scooting mode” but a “hopping mode” was suggested for membrane-bound substrate accession through ALOX15B. In this suggested mode of action, ALOX15B is thought to hop from one membrane contact point to the next, thereby reaching all available substrates. This mode requires a collision of enzyme and membrane for each turnover (Bender et al., 2016).

## LOX-mediated fatty acid peroxidation

LOX-catalyzed fatty acid peroxidation is widely recognized as the free radical mechanism (de Groot et al., 1975; Ludwig et al., 1987; Lehnert and Solomon 2003). The non-heme iron (Fe^3+^, active) in the catalytic center of the LOX enzyme promotes a stereospecific hydrogen abstraction from the central carbon of a *cis*,*cis*-1,4-pentadiene PUFA moiety, generating a carbon-centered fatty acid radical. Subsequently, the disintegration of the abstracted hydrogen atom facilitates a proton-coupled electron transfer of a single electron to the catalytic Fe^3+^ center, leading to an inactive Fe^2+^. Followed by the rearrangement of the radical, molecular oxygen is antarafacially inserted into the PUFA backbone and the corresponding mono-peroxyl radical is formed. The catalytic machinery of the enzyme is restored by a re-oxidation of Fe^2+^ to Fe^3+^ through the electron transfer from the ferrous iron to the radical, forming a fatty acid peroxy-anion that subsequently is protonated to a hydroperoxide. For 15-LOX orthologs, AA-peroxidation involves C_13_-hydrogen abstraction and introduction of oxygen [+2] at C_15_ (Ivanov et al., 2015) (Figure 1D).

## Regiospecificity and substrate orientation of ALOX15B-catalyzed oxygenation

Although the regiospecificity of ALOX15B with respect to AA oxygenation was reported simultaneously with its discovery (Brash et al., 1997), its underlying mechanisms and the conditions that regulate substrate orientation have been the content of many further studies. In general, besides the 1) access of molecular oxygen to the reacting pentadiene, two more aspects determine LOX regiospecificity and stereospecificity: 2) the PUFA carbon-chain positioning in the active site and 3) the head-to-tail orientation of the PUFA (Newcomer and Brash 2015).

(to 2) Based on various individual crystal structures of LOX enzymes, several concepts have been proposed over the years to explain the positional specificity of LOX enzymes. Initially, with the “Triad Concept” a structural model explaining the variable regiospecificity of mammalian 15-LOX orthologs was defined on the basis of three clusters of amino acid determinants (Borngräber et al., 1999; Ivanov et al., 2010). This triad of amino acids was proposed to form the bottom of the substrate binding pocket and thus, determine the depth of substrate entry and thereby the positional specificity of 15-LOX orthologs. Although confirmed for all mammalian 15-LOX orthologs (Heydeck et al., 2022), the Triad Concept was not applicable to ALOX15B and the closely related ALOX12B (Vogel et al., 2010). More recently, the existence of a U-shaped substrate binding channel that determines the position of the PUFA-pentadiene attack has been at the center of the currently most conjectured concept (Neau et al., 2009; Kobe et al., 2014). Based on the analysis of the detergent octyltetraethylene glycol ether (C8E4)-bound crystal structure of human ALOX15B, C8E4 was found to be positioned in a U-shaped conformation in the active site of ALOX15B (Figure 1C). The superimposition of AA onto the C8E4 location placed the C_13_ atom of AA adjacent to the catalytic iron of ALOX15B, thereby generating an appropriate position for hydrogen abstraction and oxygenation at C_15_ (Kobe et al., 2014). Additionally to the substrate mimic, PUFA catalysis in a boomerang-shaped fatty acid cavity was confirmed with coral 8(*R*)-LOX binding of AA under anaerobic conditions (Neau et al., 2014).

(to 3) Control of PUFA entry in head or tail orientation is regulated by highly conserved amino acids. Based on the model of a U-shaped substrate binding cavity, these amino acids determine the positioning of the fatty acid with its respective pentadiene opposite to the non-heme iron, making it suitable for the initial stereoselective hydrogen abstraction and the subsequent reaction with molecular oxygen (Newcomer and Brash 2015). The superimposition of AA onto the ligand-bound crystal structure of human ALOX15B indicated that only the entry of the substrate in a “tail first” orientation places the reactive pentadiene close to the catalytic iron of ALOX15B (Kobe et al., 2014). This concept was supported by the analysis of the generated product profile of ALOX15B-catalyzed oxygenation of phosphatidylcholine (PC)-esterified AA (Bender et al., 2016). The exclusive detection of 15(*S*)-HETE-PC indicated that fatty acids enter the active site of ALOX15B with their carbon tail first since the bulky PC head group does not allow for another orientation (Coffa and Brash 2004).

Moreover, free energy simulations confirmed the “tail first” orientation of substrate entry into the active site of ALOX15B (Suardíaz et al., 2016). Calculations indicated an abstraction of the pro-*S* H_13_ hydrogen from C_13_ of AA, therefore only allowing the “tail first” entry and the exclusive generation of 15-HpETE by ALOX15B. Interestingly, mutation of single amino acids to the corresponding amino acids in mouse Alox8 changed the “tail first” orientation of ALOX15B towards the Alox8 typical “head first” substrate entry and *vice versa* (Jisaka et al., 2000). Moreover, this amino acid exchange converted the positional specificity from 8(*S*) to nearly exclusive generation of 15(*S*) (Bender et al., 2016). Recently, these *in vitro* mutagenesis studies have been confirmed through the generation of *Alox15b* knock-in mice, in which the reported amino acids of Alox8 were substituted by those from human ALOX15B. As these humanized *Alox15b* knock-in mice formed only small amounts of 8-HETE, the substitution from Tyr^603^/His^604^ in Alox8 towards Asp^602^/Val^603^ in Alox15b confirms that these amino acid pairs are responsible for the different regiospecificity of the two enzymes (Schäfer et al., 2022).

## ALOX15B reaction specificity

**FIGURE 2:**
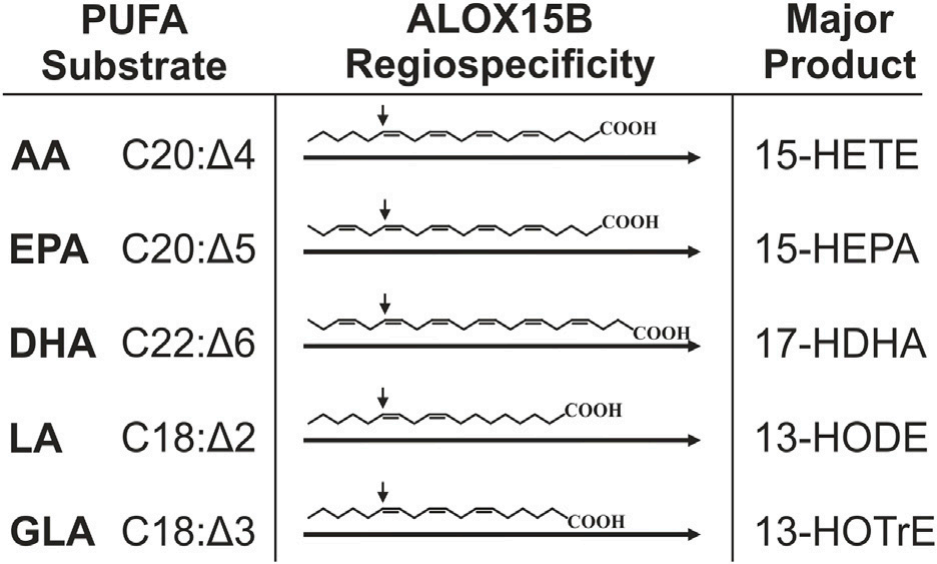
Major products derived from PUFA oxygenation through ALOX15B. Singular reaction specificity toward various PUFA substrates was demonstrated for recombinant and purified ALOX15B but also in cellular systems this was apparent. This figure summarizes major hydroperoxides generated by ALOX15B-catalyzed oxygenation of ω-6 PUFAs AA (arachidonic acid), LA (linoleic acid) and GLA (γ-linoleic acid) as well as ω-3 fatty acids EPA (eicosapentaenoic acid) and DHA (docosahexaenoic acid). AA is mainly transformed to 15-hydroxyeicosatetraenoic acid (15-HETE), EPA to 15-hydroxyeicosapentaenoic acid (15-HEPA), DHA to 17-hydroxydocosahexaenoic acid (17-HDHA), LA to 13-hydroxyoctadecadienoic acid (13-HODE), and GLA to 13-hydroxyoctadecatrienoic acid (13-HOTrE). The arrow indicates the site of *S*-stereospecific oxygen insertion into the respective PUFA mediated by ALOX15B.

Mammalian LOX enzymes are best studied for their peroxidation of free PUFA substrates including the biologically most abundant omega-6 (ω-6) PUFAs AA (C20:Δ4) and LA (C18:Δ2) as well as the less abundant ω-3 PUFAs EPA (C20:Δ5) and DHA (C22:Δ6) (Kühn et al., 1993; Kutzner et al., 2017). Although named after their concordant oxygenation of AA at C_15_ (Kühn et al., 2015), 15-LOX orthologs exhibit variable substrate reaction specificities. In contrast to human ALOX15, which was described to not only catalyze the formation of AA to 15(*S*)-HpETE but also 12(*S*)-HpETE (Bryant et al., 1982; Kutzner et al., 2017), ALOX15B exclusively produces 15(*S*)-HpETE from AA (Brash 1999; Kilty et al., 1999; Jacquot et al., 2008; Kutzner et al., 2017). Using purified recombinant enzyme preparations, singular positional specificity of human ALOX15B was also reported for EPA and DHA oxygenation and in simultaneous incubation of ALOX15B with a mixture of substrate PUFAs the singular positional oxygenation specificity of ALOX15B was maintained (Kutzner et al., 2017) (Figure 2). Moreover, a general preference of 15-LOX orthologs for PUFAs with a higher degree of unsaturation was detected in the used experimental conditions and DHA was indicated as the overall preferred PUFA substrate between AA, EPA and DHA (Kutzner et al., 2017). Product analyses of AA and LA oxygenation showed that AA was preferred over LA (Brash 1999; Kutzner et al., 2017) and LA over γ-linolenic acid (GLA, 18:Δ3) (Kutzner et al., 2017). Contrarily, also using purified enzyme, other groups reported a substrate preference of ALOX15B for LA in direct comparison with AA (Kilty et al., 1999; Wecksler et al., 2008). However, the described substrate preference of the isolated protein is reported to differ from the specificity in intact macrophages. There, 50% less 15-LOX catalyzed DHA oxygenation products were identified than monohydroxylated fatty acids derived from AA and EPA (Ebert et al., 2020). As mentioned by Ebert et al., 2020, the reaction specificity of LOXs in intact cells is potentially affected by the availability of the substrate, the substrate specificity of other lipid-mediator generating enzymes and the presence of fatty acid liberating phospholipases, which make it difficult to reconcile a clear substrate specificity of LOX enzymes in the cellular system with those of isolated enzyme.

Among all LOX enzymes, oxygenation of ester lipid-bound PUFAs (Schewe et al., 1975; Kühn et al., 1990; Coffa and Brash 2004) and lipoproteins (Belkner et al., 1993; Kühn et al., 1994; Belkner et al., 1998) is a peculiar characteristic specific to 15-LOX orthologs. In line with its reported singular reaction specificity of free substrate PUFAs, recombinant ALOX15B was found to oxygenate phospholipid (PL)-esterified AA exclusively to 15-HETE (Coffa and Brash 2004; Bender et al., 2016). Recalling the suggested Ca^2+^-dependent membrane association, membrane-bound activity of ALOX15B has been shown by incubation of exogenous ALOX15B with PUFA-enriched HEK293 lysates. Addition of exogenous phospholipase A_2_ (PLA_2_) to heat-inactivated ALOX15B massively liberated 15-HETE, pointing toward an ALOX15B-generated membrane PL-esterified product, generated from the esterified substrate. However, data from cellular systems are lacking as no conclusive discrimination between free and esterified product could be made from experiments using HEK293 cells expressing ALOX15B (Bender et al., 2016).

## Inhibition of ALOX15B

The only inhibitors known to date that specifically inhibit ALOX15B with micromolar potency were synthesized based on the concept of a U-shaped substrate binding cavity (Jameson et al., 2014; Tsai et al., 2021a). The respective inhibitors (MLS000327069, MLS000327186, and MLS000327206) are described to have a 50-fold higher selectivity for ALOX15B versus human ALOX5, ALOX12, ALOX15, cyclooxygenase (COX)-1 and COX-2, without affecting redox activity (Tsai et al., 2021a). Besides recombinant enzyme preparations, inhibition of ALOX15B was studied in a cell assay that used ALOX15B-overexpressing HEK293T cells. The cells were collected, diluted, incubated with the respective inhibitor for 20 min at 37°C, following stimulation with CaCl_2_ (100 µM), calcium-Ionophore A23487 (5 µM) and AA (1 µM). Proof of ALOX15B inhibition was assessed by measurement of 15-HETE levels. In addition to these ALOX15B-specific inhibitors, other compounds have been ascribed an inhibitory function for ALOX15B. Apart from the universal LOX and COX inhibitor nordihydroguaiaretic acid (NDGA) (Vasquez-Martinez et al., 2007; Kakularam et al., 2022), PD146176 (Abrial et al., 2015) and distinct flavonoids (Vasquez-Martinez et al., 2007; Mascayano et al., 2013) have been described to inhibit ALOX15B activity.

## Product-regulation of ALOX15B enzyme function

In normal human prostate epithelial cells (NHP), which basally express ALOX15B, Ca^2+^-induced membrane association of ALOX15B massively increased enzyme activity, possibly due to improved enzyme stabilization through membrane or substrate binding (Kilty et al., 1999). However, regulation of enzyme activity and substrate specificity of LOX enzymes has been shown to occur not only through substrate binding but also through their own hydro(pero)xide products. Incubation with 15-HETE (Kilty et al., 1999) and AA-derived hydroperoxides 15-HpETE and 12-HpETE as well as LA-derived 13-hydroperoxyoctadecadienoic acid (13-HpODE) (Wecksler et al., 2008) have been described to activate ALOX15 and ALOX15B. Mechanistically, it was suggested that the hydroperoxides increase Fe^2+^ oxidation, therefore enabling a faster transition towards the active enzyme status after substrate oxygenation (Jones et al., 1996; Solomon et al., 1997; Wecksler et al., 2008). Moreover, addition of 13-HpODE changed the substrate specificity of purified recombinant ALOX15B and ALOX15, whereas incubation with 12-HpETE solely affected ALOX15 and 15-HpETE mediated only little effects on substrate specificity of both enzymes. Since the reduced products 13-hydroxyoctadecadienoic acid (13-HODE) and 12-HETE also altered the substrate specificity, it was excluded that the change in iron oxidation mediates substrate specificity as it was proposed for enzyme activity (Wecksler et al., 2008). Based on this highly selective effect of LOX products, a role in allosteric regulation of ALOX15 and ALOX15B with direct effects on substrate specificity were proposed for 13-HpODE and 12-HpETE (Wecksler et al., 2008; Offenbacher and Holman 2020). Further research showed that binding of 13-HODE eliminated the kinetic dependency on the hydrogen bonding rearrangement in ALOX15B. Binding to the allosteric site, which was suggested to be at histidine H^627^, induced a conformational change in ALOX15B and thereby an increase in substrate specificity, likely by adjusting the enzyme to a catalytically competent state (Wecksler et al., 2009). It was concluded that very small amounts of 13-HODE shift the enzyme’s substrate specificity from AA towards LA and thereby promote a positive feedback loop of 13-HODE generation (Wecksler et al., 2009).

Further on, a pH-dependence and the implication of the PLAT domain in substrate specificity was reported for ALOX15B (Joshi et al., 2013). For example, ALOX15B was found to favor GLA at physiologically relevant pH but AA at slightly elevated pH values. Since the addition of 13-HODE induced the reaction of ALOX15B with AA but inhibited the reaction with GLA, an increased preference towards AA for both pH conditions was concluded (Joshi et al., 2013), which was supported by the findings of Kutzner et al. (2017). Of note, previous reports also demonstrated a controversial suicidal self-inactivation of LOXs through their own products. For example, 15-HpETE and 13-HpODE have been shown to mediate the inactivation of mammalian 15-LOXs (Rapoport et al., 1986; Gan et al., 1995). In this context, oxidation of LOX active site residues by radical intermediates from the catalytic fatty acid peroxidation was proposed as a potential mechanism (Hoobler et al., 2013). With regard to rabbit Alox15 it was shown that 15-HpETE induces a covalent modification of rabbit Alox15, leading to an inactive enzyme (Wiesner et al., 2003), which was confirmed later on by separation of proteolytic cleavage peptides (Kühn et al., 2005). As the molecular basis for suicidal enzyme inactivation has not been proven conclusively, the process of hydroperoxide-induced auto-inactivation remains unclear, especially for ALOX15B, where no suicidal self-inactivation has been reported so far.

Apart from the concept of allosteric regulation through LOX products, the allosteric regulation by enzyme monomers was proposed. In this concept, LOXs function as dimeric proteins with one monomer acting as an allosteric regulator of the other monomer (Ivanov et al., 2021). It was described that 13-HODE induces transient dimerization of rabbit Alox15 in aqueous solution (Ivanov et al., 2012). However, in contrast to rabbit Alox15, purified recombinant ALOX15B was mainly found in two monomeric conformers in aqueous solution but not as a dimer (Ivanov et al., 2021). So far, evidence is lacking whether ALOX15B monomers can act as allosteric regulators of the partner monomer.

## Transcriptional regulation of ALOX15B

**FIGURE 3:**
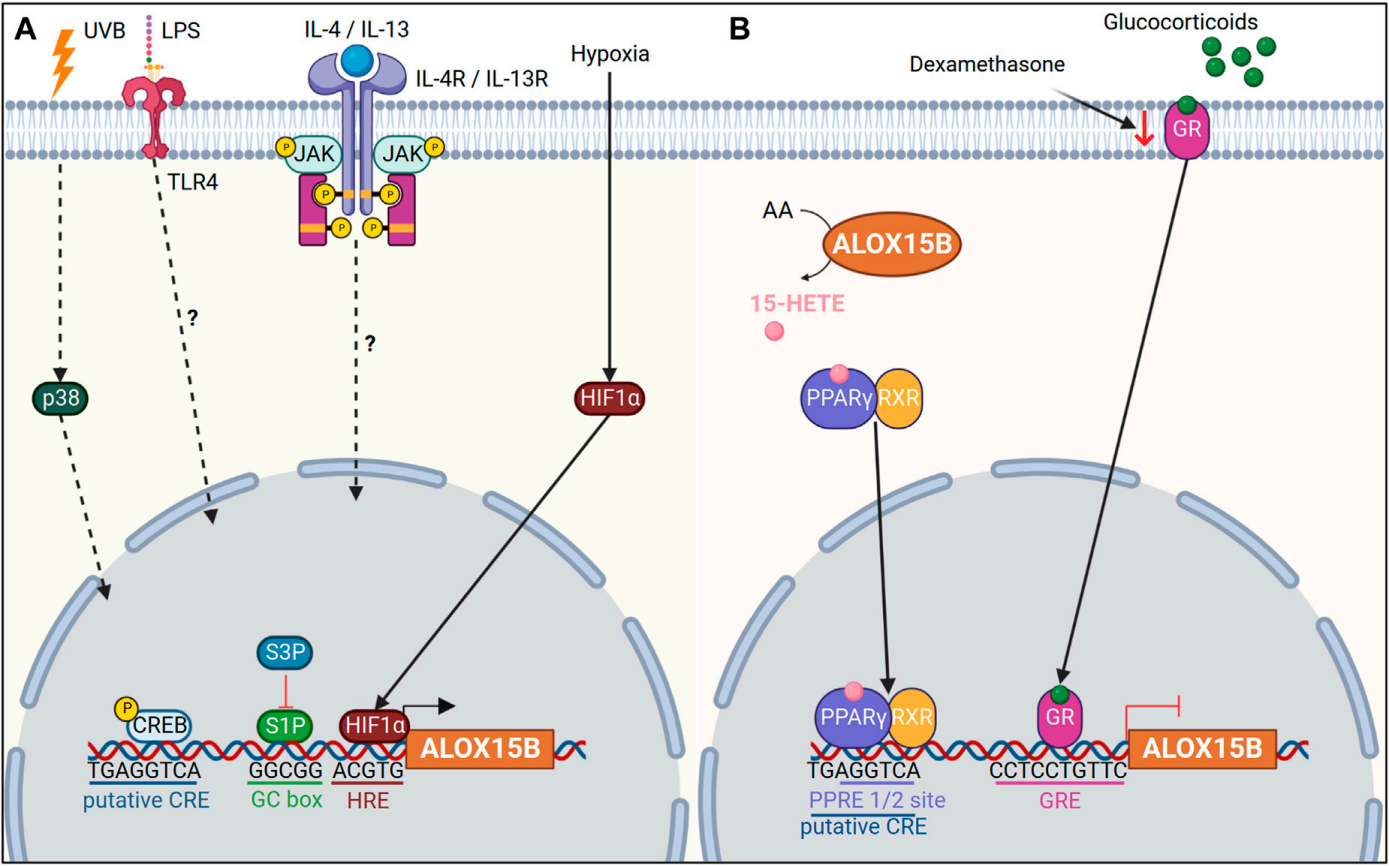
Transcriptional regulation of *ALOX15B*. **(A)** Ultraviolet B (UVB) radiation upregulates *ALOX15B* expression *via* mitogen-activated protein kinase 11 (p38). Lipopolysaccharide (LPS) and interleukin (IL)-4/IL-13 upregulate *ALOX15B* expression in macrophages *via* an unknown mechanism. Hypoxia inducible factor 1 subunit alpha (HIF1α) increases *ALOX15B* expression under hypoxic conditions. The *ALOX15B* promoter contains a putative cAMP response element (CRE) binding site, in addition to GC box binding sites for specificity protein 1 (S1P). S1P positively regulates *ALOX15B* transcription, while S3P regulates negatively. **(B)** ALOX15B activates peroxisome proliferator activated receptor gamma (PPARγ) *via* 15-HETE, which in turn negatively regulates *ALOX15B* transcription. A peroxisome proliferator response element (PPRE) half-site in the *ALOX15B* promoter overlaps with the putative CRE site. Binding of glucocorticoids to their receptor initiates binding to the glucocorticoid response element (GRE) located within the *ALOX15B* promoter, thus inhibiting expression of *ALOX15B*. Dexamethasone, a glucocorticoid, reduces expression of the glucocorticoid receptor (GR) and upregulates *ALOX15B* expression.

Transcriptional regulation of *ALOX15B* has been reported to occur through a TATA less promoter (Tang et al., 2007). However, binding sites of zinc finger transcription factors specificity protein 1 and 3 were also detected within the *ALOX15B* promoter, and have been shown to positively and negatively regulate transcription of *ALOX15B* (Figure 3A) (Tang et al., 2004).

In addition, interleukin (IL)-4/IL-13 cytokines or lipopolysaccharide (LPS) stimulation induced ALOX15B expression in macrophages (Wuest et al., 2012; Snodgrass et al., 2018). Yet, the exact mechanism of how IL-4/IL-13 or LPS induce ALOX15B remains to be uncovered. One should also keep in mind that the cytokine concentration used for enzyme induction *in vitro* differ significantly from those found under physiological conditions (Kühn et al., 2016). One potential molecular mechanism for enzyme induction seems to be through cAMP response element binding protein (CREB) signaling, mediated *via* toll like receptor 4 (TLR4) (Wen et al., 2010). In line, Subbarayan et al. (2006) reported a putative cAMP response element (CRE) in the promoter of *ALOX15B*, and treatment of NHP cells with dibutryryl cAMP increased ALOX15B expression. However, it should be noted that direct binding of phosphorylated CREB to the promoter of *ALOX15B* has not been confirmed and other nuclear receptor proteins, which are associated with IL-4/IL-13 or LPS signaling cannot be ruled out.

In human macrophages hypoxia-induced ALOX15B expression has also been reported in addition to a positive correlation of ALOX15B expression with hypoxia inducible factor 1 subunit alpha (HIF1α) in carotid plaques (Rydberg et al., 2004; Hultén et al., 2010). Furthermore, dimethyloxalylglycine, which mimics hypoxic conditions through stabilization of HIF1α, induced ALOX15B expression (Hultén et al., 2010). Likewise, ALOX15B expression was also increased under hypoxic conditions in cardiac fibroblasts (Sandstedt et al., 2018), pulmonary artery smooth muscle and endothelial cells (Liu et al., 2009), as well as lung cancer cell line A549 (Yang et al., 2018). However, in human retinal microvascular endothelial cells a reduced ALOX15B expression was observed when cells were exposed to hypoxia (Bajpai et al., 2007). *In silico* analysis of the *ALOX15B* promoter indicated three potential binding sites for HIF1α (Hultén et al., 2010) and computational analysis revealed the location of a hypoxia response element “ACGTG” immediately upstream of the transcription start site in the *ALOX15B* promoter. Yet conclusive evidence needs to be performed to see if direct binding of HIF1α to the *ALOX15B* promoter occurs or if an indirect mechanism induces ALOX15B expression under hypoxia.

As reported previously, ALOX15B products have also been shown to regulate enzyme activity (Rapoport et al., 1986; Gan et al., 1995; Kilty et al., 1999). In particular, AA-derived 15-HETE has been associated with a negative feedback loop in the regulation of the nuclear receptor peroxisome proliferator activated receptor gamma (PPARγ) (Subbarayan et al., 2006). 15-HETE, a ligand for PPARγ, was found to initiate translocation of PPARγ to the nucleus and transcription of PPARγ target genes (Sun et al., 2015). In accordance, a peroxisome proliferator response element half-site was described to be located -560 to -596 base pairs upstream of the *ALOX15B* transcription start site, overlapping with a putative CRE (Subbarayan et al., 2006). Subbarayan et al. (2006) speculated that either binding of PPARγ to the peroxisome proliferator response element half-site may therefore inhibit CREB binding to the *ALOX15B* promoter, thus inhibiting transcription of *ALOX15B* or cooperative interaction of these transcription factors may occur. Alternatively, nuclear factor kappa B (NFκB)-mediated transcription has been shown to be inhibited through PPARs (Wang et al., 2007). Although human ALOX15B has not been previously linked with NFκB, upregulation of murine Alox8 has been reported for IκBα (inhibitor of NFκB subunit alpha) deficient mice (Schneider et al., 2004).

Expression of ALOX15B has been found in the epithelium of steroidogenic tissues such as prostate (Tang et al., 2007), breast (Wang et al., 2017; Vasiliou et al., 2022), endometrium (Russell et al., 2011) and skin (Brash et al., 1997) (Table 1). Given that dysregulation in steroidogenesis is associated with cancer and other diseases, distinct and cell specific alterations in ALOX15B expression have been linked to various pathologies. Whereas treatment of breast cancer cell lines with dihydroxytestosterone (DHT) upregulated ALOX15B expression (Vasiliou et al., 2022), treatment of prostate cancer with DHT did not induce ALOX15B expression, although a partially matched androgen response element (ARE) was described to be located within ∼1.4 kb upstream of the *ALOX15B* transcription start site (Tang et al., 2004). These data correlate with the high androgen receptor activity and reduced expression of ALOX15B found in prostate cancer (Tang et al., 2007). Indeed, a glucocorticoid response element (GRE) was found within the promoter region of *ALOX15B* (Feng et al., 2010), and overexpression of the glucocorticoid receptor (GR) decreased *ALOX15B* transcription (Figure 3B). Moreover, inhibition of GR through siRNA, RU486 or dexamethasone, lowered GR expression in prostate cancer cells and restored ALOX15B expression (Feng et al., 2010). Also, expression of ALOX15B positively correlated with estrogen receptor (ER) expression in unilateral breast cancer tumors, however transcription of ALOX15B was significantly higher in ER negative breast tissue from the contralateral normal breast tissue (Wang et al., 2017). Interestingly, γ-tocotrienol, a member of the vitamin E family, has also been shown to induce ALOX15B expression (Campbell et al., 2011), whereas vitamin E treatment inhibited ALOX5 (Pein et al., 2018) and ALOX15 (Hinman et al., 2018).

## ALOX15B in inflammation and disease

### Pro-resolving lipid mediators

Regulation of inflammation is precisely controlled through diverse messengers including bioactive lipid mediators derived from LOX- or COX-mediated PUFA oxygenation (Serhan et al., 2000; Buckley et al., 2014; Schebb et al., 2022). Apart from 15-LOX generated monohydroxy fatty acids, which have been described to exhibit biological activity (Ivanov et al., 2015), 15-LOX orthologs have been implicated in the synthesis of anti-inflammatory and pro-resolving lipid mediators such as AA-derived lipoxins (lipoxygenase interaction products) (Adel et al., 2016). Lipoxins (LXs) constitute, together with EPA-derived E-series Rv, and DHA-derived D-series Rvs, protectins, and maresins the class of specialized pro-resolving lipid mediators (SPMs) (Serhan et al., 2002; Bannenberg et al., 2005; Serhan et al., 2009; Spite et al., 2009; Buckley et al., 2014; Serhan and Levy 2018). The biosynthesis of SPMs is thought to occur as a transcellular process that involves a first and a second oxygenation of the fatty acid substrate involving several LOX isoforms such as ALOX5, ALOX12, ALOX15 and ALOX15B (Schebb et al., 2022) as well as the 5-lipoxygenase activating protein (FLAP) (Lehmann et al., 2015).

The initial peroxidation of AA to 5-HpETE by ALOX5 or to 15-HpETE by ALOX15/ALOX15B forms the early precursors of LX synthesis. Using isolated and purified enzyme preparations, ALOX15B has been shown to oxygenate 5-HpETE and 5-HETE to the LX intermediate 5,15-dihydroperoxyeicosatetraenoic acid (5,15-diHpETE), which can also be generated through the ALOX5-mediated oxygenation of 15-HpETE (Green et al., 2016). Remarkably, biosynthetic *k*_cat_/*K*_M_ flux analysis revealed that ALOX15B-catalyzed conversion of 5-H(p)ETE to 5,15-HpETE happened with a 2-fold greater flux than through ALOX15 (Perry et al., 2020a). However, as ALOX15B did not further react with the LX precursor 5,15-diHpETE, no final LX generation was documented for ALOX15B (Green et al., 2016).

**FIGURE 4:**
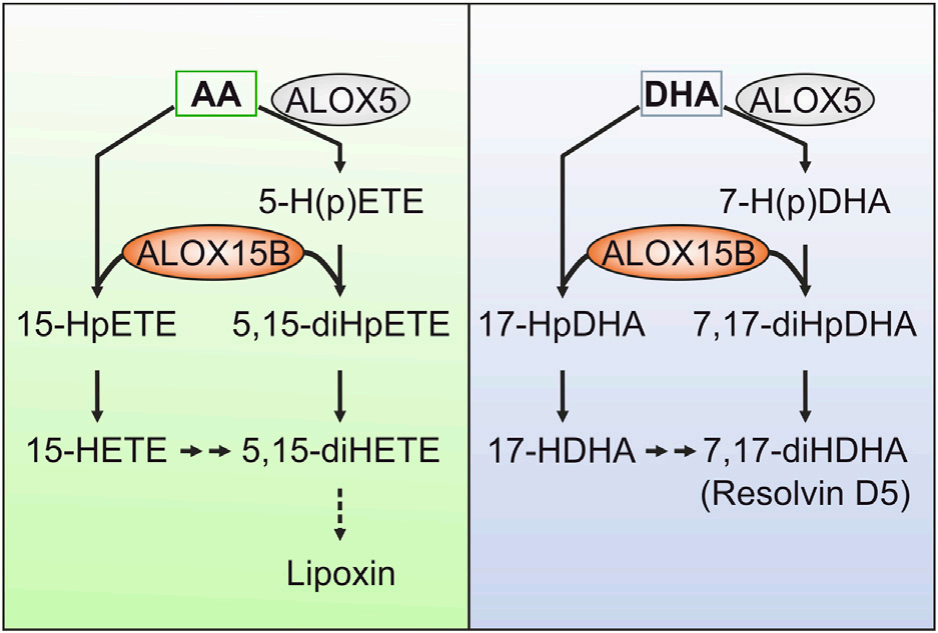
Contribution of ALOX15B in the formation of specialized pro-resolving lipid mediators (SPMs). Although debated, ALOX15B has been described to contribute to the formation of SPMs by generating mono- or dihydroxy fatty acid products that either directly perform pro-resolving functions or serve as intermediates of SPM synthesis. For example, lipoxins (LX) derive from arachidonic acid (AA), whereas docosahexaenoic acid (DHA) gives rise to resolvins (Rv). ALOX15B has been implicated in the oxygenation of ALOX5-derived AA metabolites 5-hydroperoxyeicosatetraenoic acid (HpETE) and 5-hydroxyeicosatetraenoic acid (HETE) to the LX intermediate 5,15-dihydroperoxyeicosatetraenoic acid (diHpETE). As ALOX15B did not further react with the LX precursor 5,15-diHpETE, no ultimate LX generation was attributed to ALOX15B. With respect to DHA-derived Rvs, ALOX15B was reported to catalyze the formation of the SPM precursor 17-hydroxyl-DHA (HDHA). Furthermore, ALOX15B-mediated oxygenation of ALOX5-derived 7-hydroperoxy-DHA (HpDHA) and 7-HDHA may stimulate the formation of RvD5 (7,17-diHDHA).

Similar to its implication in LX synthesis, ALOX15B was found to oxygenate 7-HDHA and 7-HpDHA almost exclusively at C_17_, forming RvD5 (7,17-diHDHA), again with faster kinetics than ALOX15 (Perry et al., 2020b) (Figure 4). In contrast to ALOX15B, peroxidation of 7-HpDHA by ALOX15 lead primarily (80%) to the product 7,14-diHDHA, underscoring the potential of ALOX15B in RvD5 formation. Based on these findings, Perry et al. (2020b) raised the question whether ALOX15B might play a larger role in SPM biosynthesis than previously thought. Although conclusive in experiments with isolated enzyme preparations or in cellular systems, the implication of ALOX15B in SPM formation is debated as well as compliance with methodological standards in the detection of SPMs (O'Donnell et al., 2021).

Polarization of macrophages to the anti-inflammatory M2 phenotype depends on the presence of the T-helper type 2 (Th2) cytokine IL-4 (Gordon and Martinez, 2010). As discussed earlier, *in vitro* stimulation of macrophages with IL-4 increased the expression of ALOX15 (Conrad et al., 1992) and ALOX15B as well as their products, 15-HETE and 13-HODE (Snodgrass et al., 2018). However, only silencing of ALOX15, but not ALOX15B, decreased the SPM precursors 15-HETE and 13-HODE to basal levels, hinting toward a small potential of ALOX15B in contribution to SPM synthesis from these hydroperoxides in primary human macrophages. Also, stimulation of macrophages with inflammatory stimuli lead to only minor detection of 15-LOX-derived lipid metabolites (Wuest et al., 2012; Russell et al., 2019; Werner et al., 2019). Given that ALOX15B is constitutively expressed in primary human macrophages and its expression is increased upon bacterial LPS treatment (Wuest et al., 2012; Snodgrass et al., 2018; Russell et al., 2019), the implication of ALOX15B in formation of SPM intermediates was questioned (Snodgrass and Brüne 2019). On the contrary, the stimulation of previously polarized M2-type macrophages with LPS induced a much higher release of 17-HDHA than of 14-HDHA (Ebert et al., 2020). For this, one has to keep in mind that it has been shown that ALOX15 generates equal amounts of 17-HDHA and 14-HDHA but ALOX15B forms almost exclusively 17-HDHA (Kutzner et al., 2017; Tsai et al., 2021b). Therefore, based on this high 17-HDHA/14-HDHA ratio in LPS-treated M2 macrophages the SPM precursor 17-HDHA is potentially primarily derived from ALOX15B (Ebert et al., 2020). Interestingly, only TLR stimulation of M2-polarized macrophages but neither of naïve nor pro-inflammatory M1-polarized macrophages led to a concerted increase of ALOX15B mRNA and protein above its basal level. In these cells, in which ALOX15 was expressed at a similar level as in control cells, the SPM intermediate 5,15-diHETE as well as RvD5 have been most abundant compared with all other conditions (Ebert et al., 2020). This is in accordance with the data of isolated ALOX15B protein that demonstrated a function of ALOX15B in the formation of the LX precursor 5,15-diHETE as well as RvD5 (Green et al., 2016; Perry et al., 2020a; Perry et al., 2020b). These data link ALOX15B to a contributing role in SPM synthesis, with a focus on the anti-inflammatory M2-type macrophages that helps to support resolution of inflammation after undergoing a priming pro-inflammatory phase. Data from tissues of children that suffer from the inflammatory lung disease cystic fibrosis suggest that decreased expression of ALOX15B is associated with low LXA_4_ levels (Ringholz et al., 2014) and that the activity of TLR4, which binds LPS, is crucial to maintain tissue homeostasis and to promote resolution of inflammation (Yang et al., 2012). Nevertheless, future studies are necessary to evaluate the role of ALOX15B-catalyzed SPM synthesis during inflammation and its resolution in man.

### ALOX15B in atherosclerosis

**FIGURE 5:**
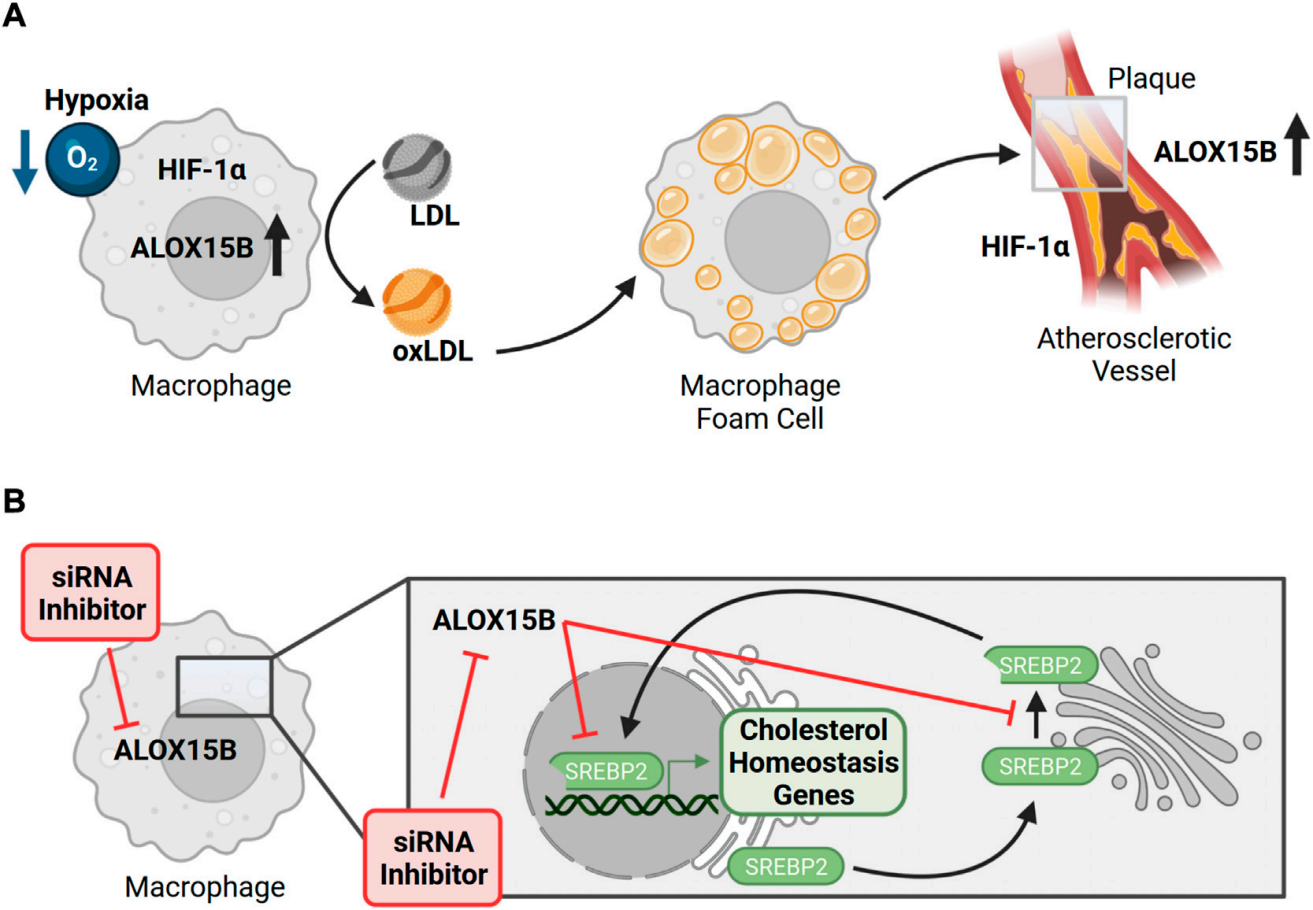
ALOX15B in atherosclerosis and its implication on macrophage cholesterol homeostasis. **(A)** Hypoxic conditions of primary human macrophages promote oxidation of low-density lipoprotein (LDL → oxLDL) and an increase of ALOX15B expression and activity. In the arterial wall, macrophages accumulate oxLDL as cholesterol esters, provoking the formation of macrophage foam cells, which can assemble into an atherosclerotic plaque. Due to the thickening of the vessel wall, reduced diffusion gives rise to hypoxic areas. Atherosclerotic plaque tissue (grey box) is associated with higher expression of ALOX15B, which correlates with the staining of macrophage-rich areas and hypoxia inducible factor 1 subunit alpha (HIF1α). While stabilization of HIF1α increases ALOX15B expression, overexpression of ALOX15B induces the secretion of chemokines and migration of T-cells, which play important roles in atherogenesis. **(B)** In primary human macrophages inhibition as well as silencing of ALOX15B impairs cellular cholesterol homeostasis by limiting maturation of the major transcription factor sterol regulatory element binding protein 2 (SREBP2) as well as its binding to target gene promotors. Therefore, suppressing ALOX15B in macrophages reduces expression of genes implicated in endogenous cholesterol biosynthesis and cholesterol uptake, which is confirmed by attenuated levels of cholesterol biosynthetic intermediates.

Atherosclerosis is a multifactorial inflammatory disease, which progressively is driven by lipid retention and inflammation. In the arterial wall, macrophages accumulate oxidized low-density lipoproteins (oxLDL) and cholesterol esters, resulting in macrophage foam cells. Due to the growing atherosclerotic plaque size and thickening of the vessel wall, impaired diffusion gives rise to hypoxic areas (<1% O_2_) and the recruitment of various immune cells supports chronic inflammation (Koelwyn et al., 2018). Culture of primary human macrophages under hypoxic conditions promoted LDL oxidation and increased ALOX15B expression and activity (Rydberg et al., 2004). In line, ALOX15B staining was enhanced in atherosclerotic plaque tissue (Rydberg et al., 2004; Gertow et al., 2011) and correlated well with the staining of macrophage-rich areas and HIF1α (Hultén et al., 2010) (Figure 5A). Interestingly, ALOX15B mRNA levels were described to be higher in symptomatic plaques with attributable cerebrovascular symptoms (amaurosis fugax, transient ischemic attack, or stroke) than in asymptomatic plaques (Gertow et al., 2011). Supporting the data of its hypoxic regulation, stabilization of HIF1α provoked ALOX15B expression and the knockdown of *HIF1α* reduced ALOX15B activity (Hultén et al., 2010). Since transcriptional activity of *HIF1α* is associated with an inflammatory plaque phenotype (Vink et al., 2007), release of pro-inflammatory signal molecules was assessed. Indeed, macrophages overexpressing ALOX15B increased the secretion of chemokine (C-X-C motif) ligand 10 (CXCL10) and C-C motif chemokine ligand 2 (CCL2) as well as activation and migration of T-cells (Danielsson et al., 2008). Accordingly, knockdown of *ALOX15B* decreased macrophage lipid accumulation as well as pro-inflammatory cytokine secretion (Magnusson et al., 2012). This was confirmed by silencing of mouse *Alox8*, which decreased plaque lipid content, plaque localized T-cell number and inflammation markers in LDL-receptor deficient mice, therefore linking ALOX15B to a pro-atherogenic role by impacting foam cell formation, immune cell recruitment and local inflammation (Magnusson et al., 2012). In line, data of a case-control study indicated the association of distinct *ALOX15B* gene polymorphisms with coronary artery disease (Wuest et al., 2014) and patients diagnosed with stroke were found to have high expression of ALOX15B (Vijil et al., 2014). Also, ALOX15B expression in human carotid plaques was associated with thrombus formation along with *in vitro* increased platelet aggregation and thrombus formation mediated by ALOX15B products 15-HpETE and 15-HETE (Vijil et al., 2014). Concurringly, knockdown of *ALOX15B* in macrophages reduced 15-HETE formation and decreased platelet aggregation and thrombin generation. Interestingly, and in contrast to 15-HETE, metabolites 7,14-diHDHA and RvD5 share potent anti-aggregation properties and therefore were proposed to directly regulate clot resolution (Perry et al., 2020b) and also 17-HpDHA demonstrated anti-aggregation properties against isolated human platelets (Tsai et al., 2021b). As all three bioactive lipid mediators were generated by ALOX15B, an ambiguous role for ALOX15B in clot formation can be assumed.

Rupture of the atherosclerotic plaque, which can clot the vessel and thereby lead to thrombosis, stroke or heart attack, is promoted by a high blood pressure. Besides its atherogenic role, ALOX15B was suggested as a driver of pulmonary hypertension (Shen et al., 2013) and has been described to induce over-proliferation of pulmonary artery endothelial cells (PAECs) (Zhang et al., 2018). In PAECs, hypoxia-induced ubiquitination of ALOX15B was proposed to initiate the sorting of ALOX15B into exosomes. Upon subsequent secretion, they were thought to trigger signal transducer and activator of transcription 3 (STAT3)-dependent proliferation of PAECs, promoting a dysfunctional and abnormal remodeling of the pulmonary vascular homeostasis. In line, hypoxia-induced expression of ALOX15B and production of its metabolite 15-HETE promoted proliferation of rabbit pulmonary artery smooth muscle cells, likely through the mitogen-activated protein kinase 3/1 (ERK1/2) mitogen-activated protein kinase (MAPK) pathway (Shan et al., 2012). Again, this sheds light on ALOX15B as a potential participant in hypoxia-induced pulmonary hypertension. In line, expression of ALOX15B was detected in fibroblasts isolated from hearts of healthy donors or donors suffering from chronic heart failure and its expression was increased upon hypoxia (Sandstedt et al., 2018). Collectively, these data demonstrate a role of ALOX15B in cardiovascular inflammation and pathophysiology. More research of ALOX15B as a driver of cardiovascular events is warranted.

### ALOX15B in cellular cholesterol homeostasis

In the pathophysiological context of atherosclerosis macrophages are confronted with the task to restore cellular cholesterol homeostasis. As phagocytes, macrophages eliminate pathogens and pathogen-infected mammalian cells and contribute to the maintenance of tissue homeostasis by eliminating apoptotic cells, named efferocytosis (Arandjelovic and Ravichandran, 2015). During cell clearance, macrophages take up immense levels of surplus lipids. To achieve cellular cholesterol homeostasis as quickly as possible, excess sterol intermediates simultaneously activate liver X receptor (LXR) target genes, which induce cholesterol export, and suppress sterol regulatory element binding protein 2 (SREBP2)-dependent genes to inhibit endogenous cholesterol biosynthesis (Spann et al., 2012; Snodgrass et al., 2021). Regarding macrophage cholesterol homeostasis, we recently reported that silencing *ALOX15B* in primary human macrophages reduces maturation of SREBP2 to an active transcription factor, binding of SREBP2 to sterol regulatory elements, and SREBP2-dependent gene expression (Snodgrass et al., 2018) (Figure 5B). In accordance, suppression of ALOX15B lowered cellular cholesterol, sterol intermediates lanosterol, 24,25-dihydrolanosterol and desmosterol as well as 27-hydroxycholesterol. During IL-4 treatment of macrophages, knockdown of *ALOX15B* and, to a lesser extent *ALOX15*, lowered the production of CCL17 in a SREBP2-dependent manner, thereby reducing the migration of CCR4 (CCL17 receptor)-expressing T-cells (Snodgrass et al., 2018). Analysis of datasets from human bronchoalveolar lavage cells isolated from control and asthma patients revealed high expression of ALOX15B, CCL17, and SREBP2-dependent genes in severe asthmatics compared with healthy donors and those with moderate asthma, implicating ALOX15B and SREBP2 in a pathophysiological relevant context.

Based on these findings, we continued to study the role of ALOX15B in macrophage cholesterol homeostasis. Based on whole transcriptome analyses and verification at both mRNA and protein levels, we confirmed our previous findings of reduced SREBP2 target gene expression in *ALOX15B*-silenced macrophages. In addition, suppression of ALOX15B disrupts intracellular cholesterol distribution in primary human macrophages. Thus, our data will add to the limited information available on the biological function of ALOX15B in primary human macrophages through modulating intracellular cholesterol homeostasis. These data may help to clarify the impact of ALOX15B expression in cholesterol-related diseases such as atherosclerosis.

### ALOX15B in inflammatory lung diseases

Recently the role of ALOX15 in inflammatory airway diseases was reviewed (Xu et al., 2021). The authors state that ALOX15 plays a greater importance than ALOX15B in the respiratory system. Indeed, a Th2 cytokine response and thus ALOX15 expression, as shown for A549 cells (Han et al., 2014), is present in the pathology of asthma. Nevertheless, Gras et al. (2012) report significantly higher *ALOX15B* expression in 3D cultures of airway epithelial cells grown at the air liquid interphase from severe asthma patients when compared to mild asthmatics, whereas ALOX15 revealed a non-significant small increase. Furthermore, 15-HETE was found to be secreted from the basolateral side of air liquid interphase experiments (Jakiela et al., 2013), which may be indicative of a function in reducing immune cell infiltration. In horses, an increase in *Alox15b* expression in inflammatory airway disease has also been reported (Ramery et al., 2015). In contrast, Planagumà et al. (2008) describe the reduction of *ALOX15B* expression in bronchoalveolar lavage cells, endobronchial lung biopsies and peripheral blood from patients with severe asthma, compared to non-severe asthma. However, significantly higher 15-HETE was secreted and present in the cellular plasma membrane of asthmatic patients (Profita et al., 2000).

In addition to type II pneumocytes, resident lung macrophages express ALOX15B (Gonzalez et al., 2004). Indeed, a correlation of 15-HETE and macrophages in sputum samples has been observed (Profita et al., 2000). Additionally, 15-LOX PUFA metabolites may also play a role in lung inflammation, as extracellular 13-HODE increased in asthmatic airways (Mabalirajan et al., 2013). Moreover, supplementation of the LA metabolite 13-HODE induced mitochondria dysfunction in bronchial epithelial Beas-2B cells (Mabalirajan et al., 2013). However, it should be noted that high concentrations (25 μM) of 13-HODE were added to these cells.

In contrast, cystic fibrosis patients showed a significant decrease in *ALOX15B* in macrophages and neutrophils of bronchoalveolar lavage (Ringholz et al., 2014). This reduction was associated with a lower ratio of LXA_4_:LTB_4_ as well as LXA_4_:IL-8, which the authors linked to impaired resolution of inflammation. Moreover, the authors suggested that reduced *ALOX15B* expression may be due to the cystic fibrosis gene CF transmembrane conductance regulator (CFTR), impairing macrophage function. Indeed, Shum et al. (2022) also report reduced ALOX15B expression in cystic fibrosis patients, although no significant reduction in 15-HETE was observed, lower LXA_4_ was apparent. As LXA_4_ was in much greater abundance than 15-HETE in these samples, it may be indicative of a predominate role of ALOX15B in metabolizing 5-HETE in these nasal polyp and mucosa samples. Furthermore, cells with F508del CFTR [a phenylalanine deletion common in cystic fibrosis patients (Nieddu et al., 2013)] demonstrated a further reduction of ALOX15B expression when compared to the WT CFTR expression in CFBE41o− bronchial epithelial cells (Shum et al., 2022). In addition, DHA supplementation in cystic fibrosis patients lowered 15-HETE in addition to LTB_4_ levels, along with decreasing the 15-HETE:17-HDHA ratio (Teopompi et al., 2019).

The contrasting ALOX15B expression levels between asthma and cystic fibrosis may be explained by the vastly different pathologies of these diseases. Indeed, both diseases have dysregulated airway epithelium, however, the increased Th2 cytokines in asthma could have potentially upregulated ALOX15B in both macrophages and airway epithelial cells. Furthermore, asthma symptoms involve shortness of breath resulting from a narrowing of the airway, whereas cystic fibrosis implicates impaired mucosal clearance (Fahy and Dickey 2010; Carlier et al., 2021). Interestingly, Teopompi et al. (2019) reported reduced levels of 15-HETE in chronic obstructive pulmonary disease (COPD) when compared to cystic fibrosis, which may be indicative of reduced 15-LOX enzyme levels. However, very few investigations into chronic inflammatory airway diseases have been conducted and further research is needed to fully understand the role of ALOX15B in the respiratory system.

### ALOX15B in inflammatory skin diseases

The importance of lipids in cutaneous biology is underscored by the function in the permeability barrier, in which PUFAs are integral components (Kendall et al., 2017; Simard et al., 2019). Indeed, ALOX12B and ALOXE3 have been shown to be crucial to corneocyte lipid envelope through incorporation of ω-hydroxy-ceramides (Zheng et al., 2011). In addition, lipids have been described to modulate cellular signaling, differentiation and inflammation (Krieg and Fürstenberger 2014). The mechanism of ALOX15B regulation in skin inflammation remains unclear, however, there are some suggestions pointing to a pro-resolution function.

Transcriptome analysis comparing normal human and psoriasis skin biopsies revealed lower *ALOX15B* expression in the disease state (Li et al., 2014), furthermore an additional study also detected a decrease in *ALOX15B* expression in both lesional and non-lesional psoriasis samples (Gudjonsson et al., 2009). However, AA and LA metabolites 15-HETE and 13-HODE were increased in both total and free form in lesional psoriasis (Camp et al., 1983; Fogh et al., 1987; Fogh et al., 1989b; Sorokin et al., 2018; Takeichi et al., 2019; Tyrrell et al., 2021). Additionally, 15-HETE was elevated in a rat imiquimod psoriasis model, which was reversed by dexamethasone (Pipper et al., 2019). In contrast, Grøn et al. (1993) reported a significant reduction of 15-HETE and 13-HODE, but it should be noted that this only taken from 0.2 mm psoriatic scales. Moreover, intralesional injection of 15-HETE both improved psoriasis and upregulated 15-HETE production (Fogh et al., 1988).

Fogh and Kragballe (2000) speculated on potential mechanisms how 15-LOX products may play a role in the resolution of psoriasis. Firstly, the incorporation of 15-HETE or 13-HODE into phospholipids may suggest a role in cellular signaling. Protein kinase C-β inhibition has been previously shown by 13-HODE incorporation into diacylglycerol (DAG) (Cho and Ziboh 1994a; Cho and Ziboh 1994b; Cho and Ziboh 1994c). Secondly, the increased levels of 15-HETE found in psoriatic plaques and lesions may inhibit the formation of LTB_4_, initiating a switch from pro-to anti-inflammatory lipid mediators (Fogh and Kragballe 2000).

Interestingly, 15-HETE was found to be increased in the dermis of skin equivalent formed from psoriatic keratinocytes when compared to skin equivalents from healthy patients. Furthermore, the elevated 15-HETE was reduced by the addition of α-linolenic acid (ALA) along with reducing epidermal thickness (Simard et al., 2022). In addition to the epidermis, production of 15-HETE was reported to be lower from the dermis of uninvolved psoriatic skin compared to normal (Kragballe et al., 1986). Indeed, ALOX15 has been reported to be absent from both the epidermal and dermal compartments of normal human skin and a low level of ALOX15B was reported to be present in the dermis. Human skin equivalents (Simard-Bisson et al., 2018) and cultured human dermal fibroblasts have also been shown to express *ALOX15* (Kubat et al., 2015). Which cells or 15-LOX enzyme contribute to the formation of 15-HETE in the dermis *in situ* remains unclear and upregulation of 15-LOX enzymes may occur from *in vitro* culture. Nevertheless, 15-HETE is associated with a positive correlation of collagen, laminin and fibronectin, and 13-HODE with collagen and laminin in human skin equivalents (Simard et al., 2022).

Although psoriasis is predominately a T-cell mediated disease, infiltration of monocytes/macrophages has been well characterized (Vestergaard et al., 2004; Kamata and Tada 2022). A significant increase in 15-HETE was detected in monocytes from patients with psoriatic arthritis (Wójcik et al., 2019). Epidermal expression of ALOX15B has been detected in normal human skin, as well as keratinocytes grown in culture (Shappell et al., 2001a; Setsu et al., 2006). Treatment of normal human epidermal keratinocytes and the keratinocyte cell line HaCaT with interferon-γ (IFNγ) (Setsu et al., 2006) or tumor necrosis factor-α (TNFα) in normal human epidermal keratinocytes (Banno et al., 2004) induced ALOX15B expression, indicating immune cell infiltration and inflammation may both induce epidermal ALOX15B in inflammatory skin diseases along with macrophage ALOX15B expression aiding in the production of oxylipins.

Alox8 expression is absent from normal murine skin, but can be induced by treatment with phorbol esters (Krieg et al., 1998; Heidt et al., 2000; Kim et al., 2005). Muga et al. (2000) generated skin specific Alox8 transgenic mice using a loricrin expression vector and these mice exhibited an increased keratin 1 (K1) expression. Although an increase in proliferation was observed, epidermal thinning was also reported. In line, 8(*S*)-HETE treatment induced K1 expression in WT keratinocytes, which was shown to be mediated by PPARα (Muga et al., 2000). Thuillier et al. (2002) demonstrated that both 8- and 15-HETE induce K1 expression in murine keratinocytes, in addition to activation of PPARγ and δ. Inhibition of LOXs with NDGA was shown to block PPRE and decrease PPARγ levels. Likewise, 13-HODE treatment in murine keratinocytes induced K1 expression, which was shown to be in a NFκB dependent mechanism (Ogawa et al., 2011). Where concentrations of 30 nM 13-HODE were enough to induce K1 and NFκB activity, this did not show significant differences in PPARγ activity (Ogawa et al., 2011). Furthermore, inhibition of NFκB with SN50 inhibited 13-HODE mediated K1 expression. As PPARγ is known to inhibit NFκB activity, it is unclear if 15-HETE induced K1 expression is mediated through a mechanism independent of NFκB, or if the PPARγ activation was insufficient to suppress NFκB activity.

In contrast, inducible *in vitro* overexpression in murine keratinocytes of both humanized ALOX15B and murine Alox8 reduced proliferation (Schweiger et al., 2007). This reduction was unrelated to apoptosis, but dependent on p38 kinase. Likewise, addition of the AA metabolites 8-HETE and 15-HETE both reduced bromodeoxyuridine (BrdU) incorporation, however no change with the addition of LA metabolites 9-HODE or 13-HODE have been found (Schweiger et al., 2007). The conflicting proliferation in these findings is likely to be underscored through differences in experimental design (*in vivo* vs*. in vitro*) and BrdU incorporation time points (30 min vs*.* 4 h). However, as differentiation is associated with reduced proliferation it could be plausible that increased keratinocyte differentiation occurs with overexpression of ALOX15B *in vitro*, yet this was not investigated*.* As both ALOX15B and Alox8 produced similar results Schweiger et al. (2007) hypothesized that both enzymes work in a similar manner, nevertheless this needs to be confirmed in a human setting to be more conclusive.

In addition to psoriasis, ALOX15B expression has been reported to increase with ultraviolet (UV) B radiation (Yoo et al., 2008). Furthermore, treatment of HaCaT cells with 13-HODE and 15-HETE reduced ALOX12 expression and proliferation, along with increasing apoptosis. Therefore, the authors hypothesized that UV therapy to induce ALOX15B expression can aid psoriasis through inhibiting hyperproliferation and production of pro-inflammatory 12-HETE. Moreover, ω-3 PUFAs have been shown to promote wound healing of sunburn in mice following UVB radiation (Meng et al., 2021), it is therefore plausible to speculate that induction of ALOX15B by UVB radiation may play a role in the protection or resolution of sunburn.

The information on the role of ALOX15B in other inflammatory skin diseases is scarce, however, increased 15-HETE levels were detected in lesional and peri-lesional atopic dermatitis skin (Fogh et al., 1989a), in addition to increases in 13-HODE in atopic dermatitis compared with healthy skin (Giménez-Arnau et al., 1997). Furthermore, the 15-LOX pathway (increased formation of 15-HETE and 15-HEPA) was upregulated in epidermal equivalents derived from atopic dermatitis patients with filaggrin mutations (Blunder et al., 2017). Interestingly, the skin science foundation bioinformatics hub indicates that increased *ALOX15B* expression is detected in lesional rosacea samples (Skin science foundation bioinformatics hub), yet so far no links to 15-LOXs or metabolites have been investigated.

Hellmann et al. (2018) investigated the role of Rvs in wound healing, here the authors demonstrated that topical application of Rvs to murine wound models resulted in enhanced re-epithelization. Furthermore, in cultured human keratinocytes ALOX15B expression was enhanced by calcium-mediated differentiation, which correlated with 17-HDHA production, proving that 15-LOXs can generate the Rv precursor 17-HDHA from DHA in keratinocytes. Proliferation was not altered, however, RvD2 promoted keratinocyte migration in a human wound model, which was shown to be mediated by the PI3K-AKT pathway. In addition to the promotion of wound healing, RvD2 decreased expression of the pro-inflammatory cytokine *IL1β* in the murine wound modal, demonstrating a role of ALOX15B in cutaneous wound healing and resolution of inflammation.

### ALOX15B in inflammatory bowel disease

Elevated levels of 15-HETE were noticed in inflamed human colon tissue (Zijlstra et al., 1992). Yet, Pochard et al. (2016) reported a slightly lower *ALOX15B* expression in Crohn’s disease patients and lower 15-HETE levels. ALOX15B expression was also significantly lower in colonic mucosa of ulcerative colitis patients (Mangino et al., 2006), while the DGLA (dihomo-γ-linolenic acid) metabolite 15(*S*)-hydroxyeicosatrienoic acid (HETrE) was significantly upregulated in the mucosa of remission colitis patients, compared to healthy controls (Diab et al., 2019). Moreover, inhibition of 15-LOXs with PD146176 lowered the body weight in a murine ulcerative colitis model, suggesting that 15-LOX metabolites play a role in the resolution of ulcerative colitis. Furthermore, 15-HETE induced expression of the tight junction protein 1 in human colorectal adenocarcinoma cells (Caco-2), along with reducing permeability and increasing transepithelial electrical resistance (Pochard et al., 2016). Indeed, 15-HETE induced ERK phosphorylation, and the 15-HETE mediated reduced permeability was facilitated through AMP-activated protein kinase (AMPK) induced tight junction protein 1 expression. Along these lines, either shRNA for tight junction protein 1 or the AMPK activator AICAR reversed 15-HETE effects on permeability. As inflammatory bowel disease is characterized by an impaired permeability barrier (Michielan and D'Incà 2015), therapies targeting ALOX15B expression may help in the resolution.

### ALOX15B in arthritis

Mao et al. (2020) detected increased Alox15b expression in a rat rheumatoid arthritis model and lipoprotein associated 15-HETE and 13-HODE were increased in the plasma of human rheumatoid arthritis patients (Charles-Schoeman et al., 2018). Treatment of synovial fibroblasts acquired from patients with rheumatoid arthritis with 15-HETE showed nuclear translocation of p68, an increase in phosphoinositide 3-kinase (PI3K)-mediated AKT phosphorylation and a reduced IκBα (inhibitor of NFκB subunit alpha) expression (Wu et al., 2012). 15-HETE mediated PI3K-signaling increased matrix metalloproteinase (MMP)-2, a protein facilitating degradation of joints. However, the authors linked this to ALOX15 expression, while ALOX15B was not investigated. Therefore, ALOX15B expression in human macrophages or tissues associated with rheumatoid arthritis still needs to be determined. In contrast, 13-HODE and 15-HETE decreased MMP-1 and MMP-3 expression in chondrocytes derived from patients with osteoarthritis (Chabane et al., 2009). The decrease in MMP was mediated by PPARγ, however, a 10-fold increase in oxylipin concentrations were used to achieve this. These observations were from different cell types and expression of both 15-LOX enzymes was reported in chondrocytes (Chabane et al., 2009), however physiologically relevant oxylipin concentrations need to be determined.

### ALOX15B in reproductive diseases

ALOX15B expression along with increased 15-HETE levels were noticed in the placenta and umbilical cord artery from pre-eclampsia patients (Wang et al., 2012; Yuan et al., 2014), which was associated with an increase in HIF1α (Yuan et al., 2014). Furthermore, ALOX15B expression was induced in human umbilical vein endothelial cells derived from patients with pre-eclampsia or when exposed to a hypoxic environment. Treatment of human umbilical vein endothelial cells with 15-HETE increased cell viability, proliferation, and cell cycle progression, whereas this effect was reduced by inhibition of LOXs with NDGA. Moreover, 15-HETE induced migration and tube formation, and therefore the authors suggested that the increase in 15-HETE/15-LOXs is involved as a compensatory mechanism of reduced angiogenesis in pre-eclampsia (Yuan et al., 2014).

Moreover, *Alox15b* expression was increased in the labyrinth zone (“site of maternal-fetal exchange”) of the placenta from rats fed a high ω-3 PUFA diet (Jones et al., 2013). Additionally, elevated 13-HODE levels were found in poly cystic ovarian syndrome (Dong et al., 2015). These results suggest potential implications of ALOX15B in additional reproductive disorders, however more evidence on expression and metabolite levels are needed for definitive conclusion.

Recently, a role for prostaglandin E2 (PGE2) in the induction of ALOX15B expression in amnion fibroblasts was reported (Zhang et al., 2022). Inhibition of protein kinase A or antagonism of the PGE2 receptor prevented PGE2-mediated ALOX15B expression. Moreover, 15(*S*)-HETE activated the NF-κB pathway through phosphorylation of p65, leading to an increased expression of COX-2 and PGE2 synthesis. Interestingly, increased 15-HETE and PGE2 levels were detected in the amnion of termed birth deliveries in which labor occurred than in births without labor. Furthermore, 15(*S*)-HETE increases with gestational age in the murine fetal membrane and placenta. Subcutaneous injection of 15(*S*)-HETE resulted in 50% of murine births to be premature. The authors hypothesize that interruption of the positive feedback loop between COX-2 and 15-LOXs could provide a therapeutic target in preventing premature births.

### ALOX15B in obesity

*ALOX15B* expression was significantly increased in both obese and lean critically ill patients in comparison to healthy controls, which correlated with increased PPARγ expression along with cellular differentiation and proliferation. However, 15(*S*)-HETE levels remained unchanged (Goossens et al., 2017). *ALOX15B* was predominantly expressed in the CD34^−^leukocyte population of adipose tissue. Although no *ALOX15B* expression was detected in the adipocyte fraction, a low level of *ALOX15B* was found in the CD34^+^ population, which contained pre-adipocytes. However, protein expression of ALOX15B was not examined in this study (Dobrian et al., 2010). In contrast, 15-HETE has been positively correlated with body mass index (BMI) and waist circumference (Pickens et al., 2017). Furthermore, Song et al. (2016) suggested 15(*S*)-HETE to be a positive regulator of proliferation and PPARγ activation in the murine pre-adipocyte cell line 3T3-L1; however, the focus here was on Alox15.

### ALOX15B in multiple sclerosis

*ALOX15B* was found to be the 3rd highest differentially expressed gene in slowly expanding lesions from progressive multiple sclerosis (Jäckle et al., 2020). Validation by immunohistochemistry revealed ALOX15B expression in macrophages/microglia at the lesion edge, along with significantly higher expression in slowly expanding lesions than in chronic inactive lesions. Safizadeh et al. (2018) also detected significantly higher *ALOX15B* expression, 15-HETE and 13-HODE in peripheral blood mononuclear cells (PBMCs) from multiple sclerosis patients. Interestingly, higher expression of 15-LOXs/13-HODE/15-HETE correlated with higher circulating cholesterol levels.

### The role of ALOX15B in cancer

**FIGURE 6:**
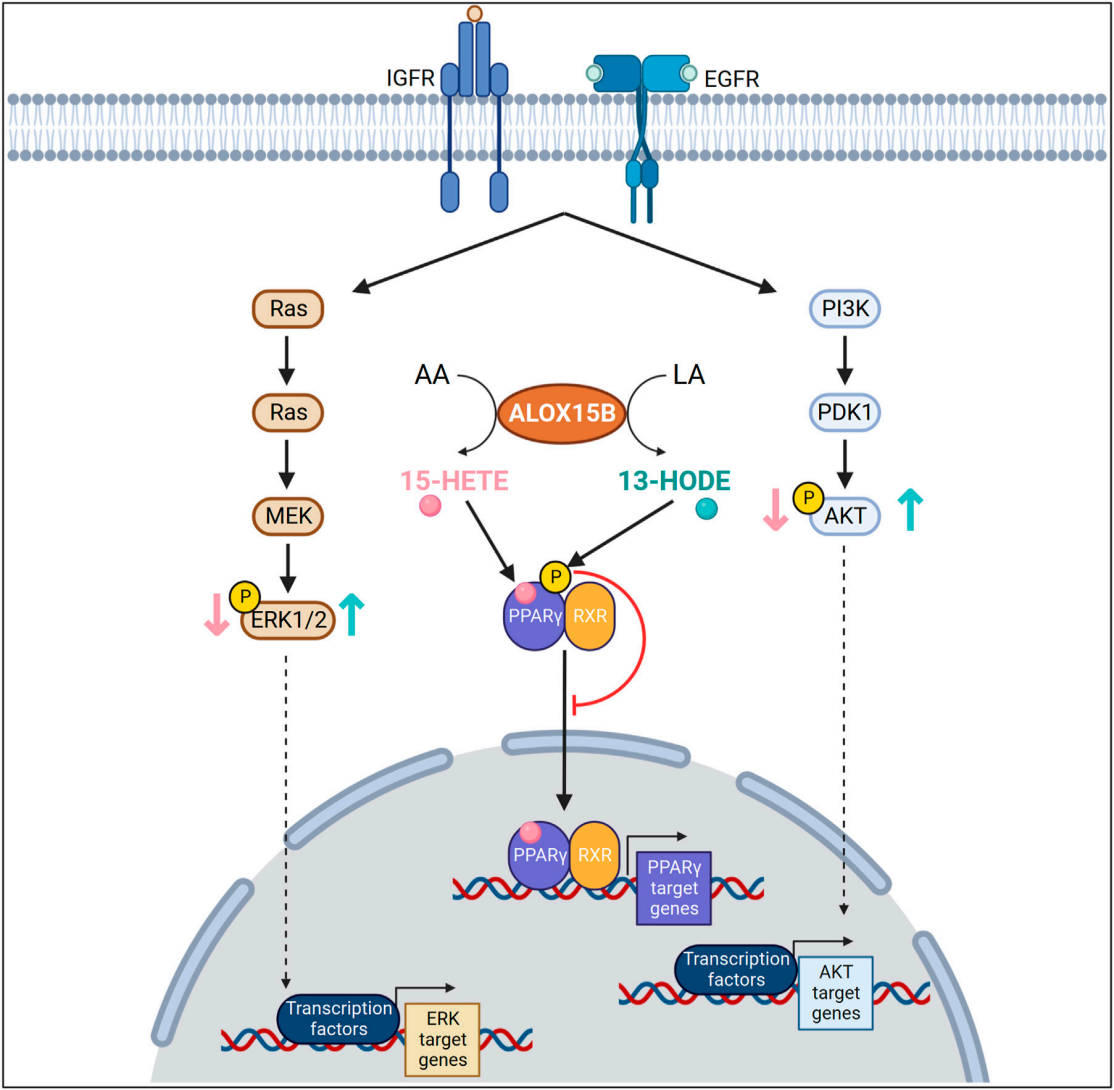
Arachidonic (AA) and linoleic acid (LA) metabolites in cellular signaling in cancer cells. ALOX15B converts AA or LA into 15-HETE or 13-HODE, respectively. In an insulin like growth factor (IGF) or epidermal growth factor (EGF) dependent manner, 15-HETE decreases mitogen-activated protein kinase 3 (ERK1), mitogen-activated protein kinase 1 (ERK2) or AKT phosphorylation, whereas 13-HODE increases their phosphorylation. 15-HETE, a known ligand for peroxisome proliferator activated receptor gamma (PPARγ) activates and increases transcriptional activity of PPARγ. In contrast, 13-HODE increases the phosphorylation of PPARγ, thereby reducing transcription of PPARγ target genes.

Investigations of ALOX15B in cancer have predominantly focused on a role as a tumor suppressor; however, conflicting evidence suggests that tissue-dependent regulation and functions are apparent. Whereas in prostate (Shappell et al., 1999; Bhatia et al., 2003; Tang et al., 2004; Suraneni et al., 2010) and breast carcinoma (Kort et al., 1992; Jiang et al., 2006; Vaezi et al., 2021; Wu et al., 2021) ALOX15B is downregulated, expression of ALOX15B in colorectal cancer is associated with a poorer prognosis (Yuan et al., 2020). Table 3 provides further insight into expression alterations of ALOX15B or its products 15-HETE and 13-HODE in different types of cancers. Moreover, research towards ALOX15B silencing or overexpression in cancer cells lines has been performed, providing further insight. The data suggest a role of ALOX15B in cellular signaling pathways (PPAR, MAPK/ERK, AKT), which are linked to cellular survival, proliferation, and apoptosis (Figure 6). Therefore, the following paragraphs will summarize the information on prostate, breast, and lung carcinomas.

**TABLE 3:** ALOX15B expression, 13-HODE and 15-HETE levels in cancer.

Cancer	Subtypes	ALOX15B expression	13-HODE	15-HETE	References
Adrenocortical	Adenoma Carcinoma	**↑** (carcinoma lower than adenoma)			Soon et al. (2008)
Breast		**↓**		**↑**	Jiang et al. (2006), Wu et al. (2021), Vaezi et al. (2021), Subbarayan et al. (2006), and Kort et al. (1992)
Bladder	Traditional cell carcinoma	**↓**		**↓**	Subbarayan et al. (2005)
Colorectal		**↑**	**↓**	No change	Yuan et al. (2020) and Shureiqi et al. (2010)
Head and Neck	Carcinoma	**↓**			Yang et al. (2008) and Wang et al. (2006)
Lung	Low cell carcinoma, Squamous cell carcinoma, Non-small cell carcinoma	**↓**	**↓**	**↓**	Li et al. (2019), Yuan et al. (2010)
	Non-small cell carcinoma, Adenocarcinoma	**↑**			Tian et al. (2016), Yang et al. (2018)
Esophageal	Squamous cell carcinoma, Adenocarcinoma	**↓**			Xu et al. (2003)
Ovarian	Carcinoma	**↑**			Reinartz et al. (2016) and Roffeis et al. (2007)
Pancreatic		Unknown		**↑**	Bodnarczuk et al. (2018)
Pituitary	Adenoma	**↑**	**↑**	**↑**	Barooni et al. (2019)
Prostate		**↓**		**↓**	Shappell et al. (2001c), Tang et al. (2002), Shappell et al. (1999), Bhatia et al. (2003), and Suraneni et al. (2014)
Renal	Renal cell carcinoma TAMs	**↑**			Daurkin et al. (2011)
Skin	Basal cell carcinoma Sebaceous carcinoma	**↓**			Shappell et al. (2001a)
Thyroid	Papillary thyroid cancer		**↓**	No change	Yang et al. (2021)

**↓** downregulated expression/reduced levels, **↑**upregulated expression/elevated levels, green indicates a potential role as a tumor suppressor; red indicates higher ALOX15B expression levels detected in cancer tissue.

While normal prostate epithelium expresses ALOX15B but not ALOX15 in the luminal compartment (Subbarayan et al., 2006), expression of ALOX15B was lost in prostate cancer (Shappell et al., 1999; Bhatia et al., 2005) as well as associated cell lines (Tang et al., 2002), and also 15-HETE production was found to be reduced (Shappell et al., 2001b). Recently Vaezi et al. (2021) reviewed the role of 15-LOXs in breast cancer, summarizing the contrasting roles of ALOX15B and ALOX15, being negative and positive respectively, towards tumor formation. This notion is reflected in prostate cancer, where ALOX15’s substrate preference to LA is reported along with differential effects of 15-HETE and 13-HODE (Hsi et al., 2002). Similar to prostate cancer, a lower expression of ALOX15B has been detected in breast cancer in comparison to normal breast tissue (Nony et al., 2005; Jiang et al., 2006; Wu et al., 2021), in addition to 15-HETE being reduced (Subbarayan et al., 2005; Vaezi et al., 2021).

While the information on prostate and breast carcinoma is relatively consistent, investigations in lung cancer have shown both increased (Gonzalez et al., 2004; Sausville et al., 2017) and decreased (Li et al., 2019) ALOX15B expression. In normal human lungs, ALOX15B is expressed in both the nucleus and cytoplasm of alveolar type II cells (Gonzalez et al., 2004). Although Gonzalez et al. (2004) reported an increase in ALOX15B expression in lung cancer, an inverse correlation with ki67 expression was observed. Furthermore, tumor grade was inversely correlated with ALOX15B (Gonzalez et al., 2004). Interestingly, ALOX15B expression was lost in neuroendocrine tumors, whereas Li et al. (2019) reported a significant reduction of ALOX15B expression in lung carcinomas when compared to non-tumor tissue. However, a normal level of ALOX15B expression was reported for the majority of adenocarcinoma samples. Differences in the reported increase or decrease in ALOX15B expression may be explained through arbitrary qualitative analysis of immunohistochemical staining.

Interestingly, significantly lower expression of ALOX15B has been reported in metastatic breast cancer (Jiang et al., 2006). Furthermore, ALOX15B was downregulated in the pre-cancerous high grade prostatic intraepithelial neoplasia (HGPIN) and had a negative correlation with Gleason score (prostate histopathological scoring guide) (Jack et al., 2000). Also, ALOX15B expression was negatively correlated with breast tumor stage and a low ALOX15:ALOX15B ratio was associated with a poorer prognosis (Jiang et al., 2006), yet amplification of *ALOX15B* had a negative effect on survival (Wang et al., 2017). A negative correlation was reported between 15-HETE and tumor diameter by Kort et al. (1992), in addition to a higher level of 15-HETE reported in malignant than benign breast tumors. As it cannot be ruled out that infiltrating macrophages or ALOX15 expressing tumor cells are a source of 15-HETE and the above-mentioned study does not directly link ALOX15B to levels of 15-HETE this could be an explanation to the increased 15-HETE levels measured. Tian et al. (2016) suggest ALOX15B may play an important role in early stage non-small cell lung carcinoma and single nucleotide polymorphisms in ALOX15B have been associated with increased risk for lung cancer (Shen et al., 2009), along with rare single nucleotide polymorphisms associated with a poorer prognosis in women with non-small cell lung carcinoma (Sausville et al., 2017). When comparing lung carcinomas with mutated tumor suppressors, ALOX15B expression was downregulated (Kim et al., 2021), whereas in adenocarcinoma both ALOX15B and 15-HETE were induced (Sausville et al., 2017; Yang et al., 2018).

In prostate carcinoma cell lines, the decrease in ALOX15B expression was neither affected by inhibition of global histone deacetylation nor by inhibition of DNA methyltransferase (Tang et al., 2002). Suraneni et al. (2014) hypothesized that overexpression of the MYC proto-oncogene in prostate cancer may account for lower ALOX15B expression. The anti-inflammatory glucocorticoid dexamethasone increased ALOX15B expression (Torosyan et al., 2006). Although dexamethasone is a ligand for the GR, treatment of prostate cancer results in the compensatory downregulation of the GR (which is overexpressed in prostate cancer). Indeed, a glucocorticoid response element is located within -157 to -33 upstream of the *ALOX15B* transcription start site, a region which was found to be critical for promoter activity (Feng et al., 2010). Therefore, GR signaling is a likely mechanism for loss of ALOX15B in prostate cancer (for more details see section “Transcriptional Regulation of ALOX15B″).

Contrastingly, DHT treatment of breast carcinoma BT474 cells increased ALOX15B expression (Vasiliou et al., 2022), whereas in prostate carcinoma LNCaP cells, transfection with a plasmid containing the ALOX15B promoter remained unresponsive to androgen treatment (Tang et al., 2004). Although Tang et al. (2004) treated LNCaP cells with 100 nM DHT, 10 nM was sufficient to induce ALOX15B expression in BT474 cells, therefore differences in concentration are unlikely to be an explanation as to the differences in DHT responses reported. As both BT474 and LNCaP cell lines were androgen responsive and expressed the androgen receptor, an indirect mechanism of DHT induction of ALOX15B in breast cancer cells is plausible. Intriguingly, ER expression inversely correlated with ALOX15B expression in normal breast tissue, however ER positive breast tumors had a higher ALOX15B expression than ER negative tumor (Wang et al., 2017). As stable ER overexpression in MCF10A cells did not significantly alter ALOX15B expression, it is likely that ER expression alone does not play a role in the transcriptional regulation of *ALOX15B* in breast cancer. BT474 cells express both ER and AR, whereas LNCaP cells do not express ER (Lafront et al., 2020), and higher androgen receptor expression was detected in ER+ breast tumors (Kensler et al., 2019). Therefore, co-expression and crosstalk of both nuclear receptors may be required for DHT to induce ALOX15B expression and explain differences in the correlation between ALOX15B and ER between normal and cancerous breast tissue.

Studies in carcinoma cell lines reveal more insight into the role of ALOX15B as a tumor suppressor in cancer through cellular signaling pathways (Figure 6). As discussed earlier, ALOX15B products may work as PPARγ ligands and 15-HETE increased transcriptional activity of PPARγ and caused expression of the fatty acid binding protein (FABP) in PC3 cells (Shappell. et al., 2001c). 15-HETE treatment of PC3 cells reduced phosphorylation of PPARγ in the presence of insulin like growth factor-1 or epidermal growth factor, therefore implying a MAPK dependent pathway (Hsi et al., 2002). Although, 13-HODE has also been reported as a PPARγ ligand (Nagy et al., 1998), 13-HODE was described to induce PPARγ phosphorylation (Hsi et al., 2002) thus, suppressing transcriptional activity (Burns and Vanden Heuvel 2007). Furthermore, overexpression of PPARγ lowered ALOX15B expression (Subbarayan et al., 2004), whilst PPARγ activation has been shown by overexpression of ALOX15B (Li et al., 2015). Likewise with prostate cancer an inverse correlation between PPARγ and ALOX15B expression has been shown in breast cancer (Subbarayan et al., 2005). Elevated levels of PPARγ in breast cancer tumors may be responsible for low ALOX15B expression. Addition of white tea extract (immature, unopened buds of *Camellia sinensis*) to lung carcinoma A549 cells upregulated ALOX15B expression along with the production of 15-HETE, which led to an upregulation of PPARγ (Mao et al., 2010). This increase in PPARγ was suppressed by lipoxygenase inhibition (*via* NGDA or caffeic acid). Moreover, inhibition of LOXs or PPARγ resulted in a reduction in white tea extract induced caspase activity, suggesting that ALOX15B induces apoptosis in A549 cells via 15-HETE mediated activation of PPARγ (Mao et al., 2010).

Activation of p38 MAPK was observed through overexpressing ALOX15B in MDA-MB-435 cells (Nony et al., 2005). Whereas addition of 15-HETE or AA to PC3 cells reduced both ERK1/2 and AKT phosphorylation, treatment with 13-HODE or LA induced their phosphorylation in an epidermal growth factor or insulin like growth factor dependent manner (Hsi et al., 2002). However, since siRNA for ALOX15 and not ALOX15B inhibited DHA induced phosphorylation of AKT or phosphoinositide-dependent kinase-1 (PDK1), Hu et al. (2013) reported that PDK1/AKT activation in PC3 cells is ALOX15 but not ALOX15B dependent, which express low levels of both enzymes. As there is a lower level of ALOX15B in PC3 cells than NHP, further reduction by siRNA may be redundant. Repetition of these experiments in NHP or ALOX15B overexpressing cells would be more accurate in understanding whether ALOX15B alters the AKT pathway.

Indeed, earlier studies in prostate cells suggested that ALOX15 favored LA to produce 13-HODE in comparison to ALOX15B predominantly metabolizing AA to 15-HETE, which stimulated the assumption that the effects of exogenous 15-HETE treatment to prostate cells correlates with ALOX15B. However, as there is an upregulation of ALOX15 in prostate carcinoma (Kelavkar et al., 2006) and both enzymes are capable of metabolizing AA and LA it is difficult to distinguish function between the two enzymes. There is a lack of investigations involving DHA treatment in an ALOX15B context and very few studies have investigated other PUFAs. Collectively these alterations in signaling pathways suggests a role for 15-HETE produced by ALOX15B as a tumor suppressor through reducing cellular proliferation, promoting apoptosis and enhancing cytokine secretion. Furthermore, this is reflected by investigations summarized in the following paragraphs.

Prostate carcinoma cells grown *in vitro* retain a low ALOX15B phenotype and do not produce 15-HETE (Shappell et al., 2001c; Bhatia et al., 2005). ALOX15B expression and hence, generation of its metabolite 15-HETE, negatively affected cellular proliferation in prostate cells. Furthermore, overexpression of ALOX15B in UMSCC1 cells (Squamous cell carcinoma derived from the oral cavity) also reduced cellular proliferation (Wang et al., 2006). Addition of 15-HETE to NHP cells reduced proliferation (Tang et al., 2002) and increased the number of cells in the G0/G1 phase (Shappell et al., 2001c). In addition, overexpression of a GFP-ALOX15B in prostate cancer cell lines DUP145 and PC3 increased the number of cells in G0/G1 and reduced those in S phase, although no significant change in the number of ki67+ cells were detected (Tang et al., 2009). Moreover, Tang et al. (2002) reported that overexpression of ALOX15B reduced the number of BrdU+ cells. Serum starving induced ALOX15B expression and decreased BrdU incorporation, which was reversed by the re-addition of serum (Tang et al., 2002). Bhatia et al. (2005) observed that overexpression of either a fully active ALOX15B, or a non-functioning splice variant decreased proliferation. Moreover, attenuated cell proliferation with ALOX15B being overexpressed correlated with increases in cellular senescence. Furthermore, both ALOX15B and 15-HETE increased with cellular passage in NHP cells (Bhatia et al., 2005). Controversially, slower proliferating NHPrE1 cells (spontaneously immortalized NHP cells) expressed lower levels of ALOX15B than the faster proliferating BHPrE1 cells (spontaneously immortalized benign human prostate cells) (Jiang et al., 2010). Suggestively, reduced proliferation is more likely to be associated with cellular senescence than ALOX15B expression itself. Future studies should investigate whether inhibition or attenuation of ALOX15B expression in NHP cells impedes the cell’s ability to senesce with increasing passage number. The ALOX15B-generated DHA metabolites 17-HDHA, 17-HpDHA along with double peroxidized products 10(*S*),17(*S*)-diHDHA (protectin DX) and RvD5, inhibited proliferation at much lower concentrations than 15-HETE in PC3, DU145 and LNCaP cells (O'Flaherty et al., 2012).

In line with these data Li et al. (2015) reported reduced proliferation and increased caspase 3 and 9 activation with 15-HETE and 13-HODE treatment in lung adenocarcinoma, NCI-H23, and large cell cancer, NCI-H460, cells. Moreover, overexpression of both 15-lipoyxgenating enzymes also induced the cleavage of caspase 3 and 9 during simultaneous treatment with both LA and AA (Li et al., 2015). Furthermore, siRNA mediated knockdown of ALOX15B reduced grape seed procyanidin extract induced cell death of A549 and EAS-2B-CSC 1198 cells (Mao et al., 2016). The authors note that although there was no increase in ALOX15 or ALOX15B expression, there was an increase in 15-HETE production, which likely resulted from a shunting of AA metabolism towards 15-LOX enzymes as grape seed extract inhibits COX-2. In a previous study Mao et al. (2010) had observed increased *ALOX15* and *ALOX15B* expression from green and white tea extracts in A549 cells. The white tea extract induced 15-HETE secretion and caspase 3 activity. Li et al. (2019) reported that smoking carcinogen 4-methylnitrosamino-l-3-pyridyl-butanone and air pollutants drive carcinogenesis though inhibition of ALOX15B expression. It was suggested that microRNAs 17, 106, 590-3p, 20a, 20b, 93 may be the likely cause of the reduced ALOX15B expression in lung cell lines Bet1A and NCI-H23 exposed to air pollution or smoking carcinogen (Li et al., 2019).

Yet in lung adenocarcinoma A549 cells, ALOX15B expression and 15-HETE production has been induced under hypoxia (Yang et al., 2018). Contrastingly, addition of 15-HETE under both normoxia and hypoxia induced proliferation and migration of A549 cells. It should be noted that this study treated the cells with 1 µM 15-HETE for 24 h, whereas Li et al. (2015) saw reductions in proliferation with 10–70 µM 15-HETE over periods of 24, 48, and 72 h, which may explain the differences observed. Additionally, 15-HETE induced STAT3 phosphorylation and inhibition of STAT3 activation prevented 15-HETE mediated proliferation and migration (Yang et al., 2018). Moreover, ubiquitination of ALOX15B and expression in exosomes activated STAT3 in hypoxic pulmonary artery endothelial cells (Zhang et al., 2018). Tumor hypoxia is extremely common and is negatively correlated with survival. Evidence in A549 cells suggested that ALOX15B promotes proliferation through 15-HETE in lung adenocarcinomas. The underlying mechanism between ALOX15B and STAT3 activation remains unknown. Future research should question if pharmaceutical inhibition of ALOX15B can be targeted in treatments for lung cancers with high ALOX15B expression.

While the ALOX15B corresponding murine ortholog Alox8 is not expressed in murine prostate glands (Shappell et al., 2003), humanized ALOX15B overexpression studies have been performed *in vivo.* Prostate carcinoma PC3 cells (which do not express ALOX15B) were injected into the mouse prostate to develop tumors and stable ALOX15B overexpression resulted in significantly smaller tumors (Bhatia et al., 2003). An additional study using GFP tagged ALOX15B overexpressing prostate carcinoma cells (DU145 and PC3) that were injected into the right flank of nude mice also resulted in a lower tumor volume (Tang et al., 2009). Furthermore, these tumors showed more cleaved caspase 3 and high numbers of apoptotic cells even though there also was a small increase in ki67+ proliferating cells (Tang et al., 2009). Tang et al. (2009) also observed a downregulation of vascular endothelial growth factor (VEGF), suggesting that ALOX15B expression may reduce angiogenesis in prostate carcinoma. Likewise Suraneni et al. (2010) reported a similar phenotype as prostate specific transgenic expression of ALOX15B caused hyperplasia, increased ki67+ cells and higher numbers of both luminal and basal cells, along with an increase of p27, a senescence marker (Suraneni et al., 2010). ALOX15B expression was associated with the age of prostate tissue (Bhatia et al., 2005). As benign prostatic hyperplasia is correlated with age, there may be an associated link of ALOX15B and hyperplasia in the human prostate. Using an enzymatically inactive splice variant of ALOX15B revealed that the PUFA-metabolizing activity of ALOX15B was integral to the tumor suppressor function in prostate carcinoma. Suraneni et al. (2014) demonstrated that although hyperplasia was noticed in ALOX15B transgenic mice, when crossed with tumor suppressor p53^−/−^ or p53^−/+^ mice, no prostate cancer occurred. Furthermore, MYC induced carcinoma was inhibited by ALOX15B expression, which is likely through induction of cellular senescence as demonstrated by p27 and β-galactosidase staining (Suraneni et al., 2014).

Treatment with both AA and 15-HETE without ALOX15B overexpression increased cell adhesion of MDA-MB-435 cells to collagen (Nony et al., 2005). The 13-HODE/15-HETE ratio inversely correlated with the level of E-cadherin expression in breast cancer MCF-7 cells as well as in colon cancer HRT-18 cells (Pasqualini et al., 2003). However, it should be noted that MDA-MB-435 cell line has been re-described as a melanoma cell line (Prasad and Gopalan, 2015). Therefore, the assumption that 15-HETE prevents migration and metastases should be applied as a general cancer principle rather than being specific for breast cancer.

As reviewed previously by Weigert et al. (2018) tumor associated macrophages (TAMs) may play a major role in the expression of ALOX15B and lipid mediation of the tumor microenvironment. Reinartz et al. (2016) observed a slight but non-significant higher level of *ALOX15B* expression in TAMs, when compared to ovarian carcinoma tumors. Indeed Roffeis et al. (2007) revealed an increase of *ALOX15B* expression in ovarian tumors in comparison to normal ovarian tissue, however the precise cell type that expresses *ALOX15B* was not determined. It is therefore possible that an increase in ALOX15B expression occurs in both in carcinoma cells derived from ovarian tissue along with TAMs.

Extremely low level of ALOX15B expression has been found in normal kidney tissue. However, expression and 15-HETE levels were increased in carcinoma tissue and TAMs. Inhibition of LOXs through NDGA in TAMs obtained from renal cell carcinoma reduced the expression of CCL2 and IL-10 (Daurkin et al., 2011). Furthermore, inhibition of 15-LOXs *via* ML351 or PD146176 in lung macrophages derived from normal lung tissue of patients undergoing surgery for carcinoma reduced cytokine and chemokine release (Abrial et al., 2015). Both IL-4/IL-13-induced release of cytokines CCL13, 18 and 22 along with LPS-induced release of TNFα, CCL2, 3 and 4, and CXCL1, 8 and 10 were reduced by 15-LOX inhibition.

Collectively, these findings suggest a role of ALOX15B acting as a tumor suppressor in breast and prostate cancer. Whereas *in vitro* experiments pointed to reduced cellular proliferation, migration and cell cycle progression along with increased apoptosis, hyperplasia *in vivo* is accompanied by attenuated tumor formation, and both *in vitro* and *in vivo* experiments link ALOX15B expression with senescence. Inhibition of MAPK and ERK signaling by ALOX15B derived 15-HETE implies an anti-tumorigenic effect. Yet, there have been no follow-up studies on the role of ALOX15B in prostate cancer since 2014. Indeed, in recent years, treatments of prostate cancer vastly improved towards androgen receptor targeted approaches. It would therefore be interesting to explore whether current therapies result in the re-expression of ALOX15B in the prostate.

## Conclusion

As a member of the heterogeneous family of lipoxygenases, ALOX15B oxygenates poly-unsaturated fatty acids with positional specificity to the corresponding hydroperoxides, which can serve in the formation of SPMs. Present in a variety of different tissues, ALOX15B expression and activity has the ability to affect many cellular signaling pathways, involved with proliferation, survival, and cell cycle. For instance, in primary human macrophages, a role of ALOX15B in regulating cholesterol homeostasis was proposed. Moreover, attenuation or inhibition of ALOX15B has been shown to reduce cytokine and chemokine production and ALOX15B expression was linked to atherosclerosis. A clear role of ALOX15B as a tumor suppressor in prostate and breast cancer is apparent, yet there remains diversity in other carcinomas. Future investigations into standardizing physiologically relevant concentrations of lipids *in vitro* may help elucidate discrepancies. Moreover, the use of cells naturally expressing ALOX15B could provide a useful tool in detecting PUFA favorability and metabolite synthesis.

## Glossary

AA: arachidonic acid
ALOX: arachidonate lipoxygenase
Alox8: arachidonate 8-lipoxygenase (murine)
AR: androgen receptor
BrdU: bromodeoxyuridine
CCL: C-C motif chemokine ligand
CFTR: CF transmembrane conductance regulator
COX: cyclooxygenase
CRE(B): cAMP response element (binding protein)
DHA: docosahexaenoic acid
DHT: dihydroxytestosterone
diHpETE: dihydroperoxyeicosatetraenoic acid
EPA: eicosapentaenoic acid
ER: estrogen receptor
ERK1/2: mitogen-activated protein kinase 3/1
GLA: gamma-linolenic acid
GR: glucocorticoid receptor
HDHA: hydroxydocosahexaenoic acid
HETE: hydroxyeicosatetraenoic acid
HIF1α: hypoxia inducible factor 1 subunit alpha
HODE: hydroxyoctadecadienoic acid
HpETE: hydroperoxyeicosatetraenoic acid
HpODE: hydroperoxyoctadecadienoic acid
IL: interleukin
K1: keratin 1
LA: linoleic acid
LOX: lipoxygenase
LPS: lipopolysaccharide
LT: leukotrienes
LX: lipoxin
MAPK: mitogen-activated protein kinase
MMP: matrix metalloproteinase
NDGA: nordihydroguaiaretic acid
NFκB: nuclear factor kappa B
NHP: normal human prostate cells
PAEC: pulmonary artery endothelial cells
PLAT: polycystin-1-lipoxygenase α-toxin
PPAR: peroxisome proliferator activated receptor
PUFA: polyenoic fatty acid
Rv: resolvin
SPM: specialized pro-resolving lipid mediator
SREBP2: sterol regulatory element binding protein 2
STAT: signal transducer and activator of transcription
TAM: tumor associated macrophage
UV: ultraviolet
(ox)LDL: (oxidized) low-density lipoprotein
